# The curious family of cytochrome P450 4F fatty acid ω-hydroxylases: recent developments in their function and relevance for human health

**DOI:** 10.1098/rsob.250115

**Published:** 2025-08-27

**Authors:** Brisa Caroline Alves Chagas, Huiting Jia, Bjoern Brixius, Simone Brixius-Anderko

**Affiliations:** ^1^ Department of Pharmaceutical Sciences, University of Pittsburgh School of Pharmacy, Pittsburgh, PA, USA

**Keywords:** cytochrome P450, fatty acid ω-hydroxylases, arachidonic acid, metabolic diseases

## Abstract

Cytochrome P450 enzymes (CYP, P450) are indispensable for human health and catalyse a plethora of reactions contributing to the clearance of xenobiotics, steroid and bile acid biosynthesis, and the functionalization of vitamins in the human body. The family of CYP4F fatty acid ω-hydroxylases consists of five isoforms, CYP4F2, CYP4F3A, CYP4F3B, CYP4F11 and CYP4F22, which share a high amino acid sequence similarity but assume different physiological functions in humans. While CYP4F2 and CYP4F3B are the major enzymes producing a lipid mediator from arachidonic acid, CYP4F3A inactivates leukotriene B4 as anti-inflammatory response, CYP4F11 is capable of effectively metabolizing drugs, and CYP4F22 contributes to a healthy skin barrier. In this review, we provide an overview of recent developments in CYP4F function and discuss potential future directions underlining their growing relevance in human health and disease.

**Figure:**
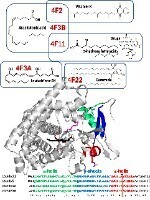

## 1. Introduction

Cytochrome P450 enzymes (P450, CYP) are heme–thiolate proteins that are conserved in all domains of life. In humans, the 57 different isoforms are indispensable for steroid hormone biosynthesis, drug and bile acid metabolism, and vitamin functionalization [1]. The family of CYP4F enzymes are fatty acid metabolizing P450 enzymes catalysing the hydroxylation of long- to ultra-long-chain fatty acids [2]. Among the CYP4F family, the isoforms CYP4F2, CYP4F3A, CYP4F3B, CYP4F11 and CYP4F22 are known as fatty acid ω-hydroxylases. They insert a hydroxyl group at the terminal carbon atom which leads to the production of various lipid mediators involved in pro- and anti-inflammatory responses, blood pressure regulation, angiogenesis and cell proliferation. Although these isoforms share a high amino acid sequence identity, they all assume slightly different functions. In addition, most are also capable of metabolizing non-fatty acid substrates which determines their biological function as a highly diverse family of enzymes. In the following, we will shed light on CYP4F commonalities and will elaborate on single isoforms and their role in human health and disease.

### 1.1. CYP4F origin

CYP4F ω-hydroxylases are located in the endoplasmic reticulum and are approximately 60 kDa in size. An N-terminal hydrophobic helix anchors them to the endoplasmic reticulum membrane with the catalytic domain exposed to the cytosol [3]. Here, they act as class I P450 redox system together with their redox partner protein nicotinamide adenine dinucleotide phosphate (NADPH)-dependent cytochrome P450 reductase (CPR) [4]. CPR is a membrane-anchored flavoprotein with a flavin mononucleotide (FMN) and a flavin adenine dinucleotide (FAD) domain [5]. To start catalysis, the CPR FAD domain abstracts two electrons from NADPH. Subsequently, the FMN domain shuttles one electron at a time to the P450 heme to perform the hydroxylation reaction.

The genes encoding the CYP4F enzymes are tandemly arranged on chromosome 19. The CYP4F family evolved from a common CYP4F ancestor during the whole genome duplication events in early vertebrates and gave rise to CYP4F22 and all other CYP4F isoforms [6,7]. The ability to metabolize fatty acids was crucial for developing life on Earth underlining the importance of CYP4F evolution. Intriguingly, a recent study focusing on the evolution of the CYP4F gene cluster reports that CYP4F enzymes evolved under balancing selection which maintains multiple versions of a gene for genome diversification [8]. This is in stark contrast to the CYP3A family evolution which are the major drug metabolizing enzymes in humans. Here, a positive selection led to specialization of one dominant isoform which is CYP3A4/5 in humans. Indeed, multiple isoforms of CYP4F enzymes are needed to maintain the body function of humans today.

### 1.2. CYP4F function

The common evolutionary origin of CYP4F ω-hydroxylases leads to a fascinating combination of joint enzyme functions but also unique isoform-specific properties. CYP4F2, CYP4F3B and CYP4F11 hydroxylate long-chain fatty acids with arachidonic acid as one of the most important substrates [9–11]. Arachidonic acid is metabolized to the lipid mediator 20-hydroxyeicosatetraenoic acid (20-HETE) which regulates the blood pressure and promotes angiogenesis [12]. CYP4F2 is mainly expressed in liver and kidney and plays a major role in vitamin K and E metabolism [13]. The CYP4F3 isoforms CYP4F3A and CYP4F3B originate from the same gene due to alternative splicing events and the insertion of two different exons, respectively [11]. CYP4F3B is a major 20-HETE producer in the liver. CYP4F3A is exclusively expressed in monocytes and contributes to the anti-inflammatory response via deactivation of leukotriene B4. CYP4F11 is unique among the CYP4F family due to its ability to efficiently metabolize drugs and 3-hydroxy fatty acids [14,15]. CYP4F22 is unlike any other CYP4F isoforms with a crucial role in the maintenance of a healthy skin barrier due to its contribution to ceramide production [16]. Recently, it has been established that CYP4F make up to 15% of all liver cytochrome P450 enzymes, the third largest group of P450 enzyme families in the liver [17]. However, of all liver P450 enzymes, the CYP4F family is the most understudied.

**Figure 1.**
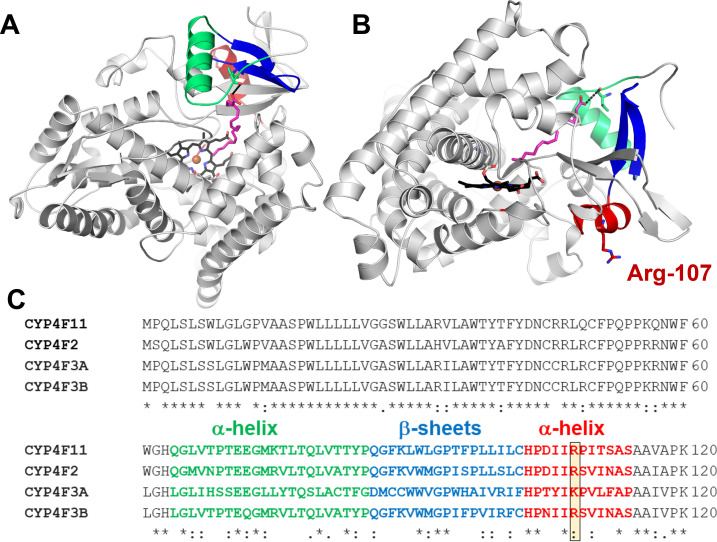
The molecular model of CYP4F11 with arachidonic acid (magenta, from [18]) (A,B) and a corresponding amino acid alignment between CYP4F isoforms (C) reveals that amino acid residues 67–114 (coloured) form an α-helix following the N-terminal anchoring helix (green) and β-sheets (blue) which both affect the substrate access channel. In red is an α-helical structure on the proximal P450 site with Arg-104 shown in sticks (boxed) which might be crucial for the interaction with redox partner proteins below the heme prosthetic group.

The CYP4F ω-hydroxylases share between 65% and 95% amino acid sequence identity. CYP4F2 and CYP4F11 are the most similar in terms of sequence. CYP4F3A largely deviates from all other isoforms. An alignment of CYP4F2, the CY4F3 variants and CYP4F11 reveals a greatly conserved amino acid sequence except for a region spanning amino acids 67−114. Interestingly, this amino acid stretch coincides with the region in CYP4F3 splice variants that is affected by different exon insertions (figure 1C). Among all isoforms, residues 67−114 exhibit the highest sequence variability [11,19]. Thus, it is reasonable to assume that those residues lead to distinct protein structures favouring some substrates over others. However, there is no structural information available for any of the human CYP4F ω-hydroxylases.

### 1.3. CYP4F structure

The only structure of a P450 ω-hydroxylase is the X-ray protein structure of rabbit CYP4B1 which catalyses the hydroxylation of short-chain fatty acids [20,21]. Even though species differences are undeniable, we gained valuable information from this structural work. The heme in P450 enzymes is usually only covalently coordinated to a highly conserved cysteine residue forming a heme–thiolate bond with the heme iron. In ω-hydroxylases, there is evidence for an additional covalent bond between the 5-methyl group of the heme protoporphyrin and a conserved glutamate residue in the I helix which makes one wall of the active site. The rabbit CYP4B1 crystal structure provides further evidence for this additional bond. It is assumed that the bond is crucial for catalysis of the energetically disfavoured ω-hydroxylation. This hypothesis is supported by mutagenesis studies which show that exchanging the conserved glutamate to alanine leads to a loss of selectivity towards ω-hydroxylation and the occurrence of an energetically more favoured ω-1-hydroxylation. The mutation results in a disruption of the covalent bond [20]. An alignment of CYP4F ω-hydroxylases indicates the presence of a glutamate in all isoforms discussed here. Despite this crucial joint feature, the lack of structural studies impedes our understanding of different catalytic functions and substrate specificity. A recently published computational model of the more promiscuous isoform CYP4F11 allows one to visualize the region spanning amino acid residues 67−114 in the protein structure with surprizing results (figure 1A,B). The residues span an α-helix and β-sheets (figure 1A,B, blue, green) located at the substrate entrance channel which is thought to be embedded into the membrane [18]. This region also contains a substrate recognition site [20]. Here, differences between the CYP4F isoforms may control the entrance and recognition of distinct substrates and affect different CYP4F body functions. In addition, residues 67−114 affect a region at the proximal P450 site which is exposed to the cytosol and crucial for the interaction with redox partner proteins (figure 1A,B, red) [22]. It is striking that the CYP4F isoforms share a conserved positively charged lysine or arginine residue at the putative redox partner binding site which could form electrostatic interactions with negatively charged residues on the redox partner surface [22]. Redox partner proteins have recently gathered attention as allosteric modulators of substrate binding and catalysis [23–25]. Thus, differences in amino acid sequence affecting the redox partner interaction site may lead to distinct allosteric regulation of catalysis and electron transfer efficiency between CYP4F isoforms.

In the following, we will provide a comprehensive overview of the five major CYP4F ω-hydroxylases, including CYP4F2, CYP4F3A, CYP4F3B, CYP4F11 and CYP4F22. We will discuss isoform-specific protein function in the human body, including current knowledge on regulation of expression and discuss clinical implications.

### 2. CYP4F2—a metabolizer of vitamins and fatty acids

#### 2.1. Discovery and substrate scope

**Figure 2.**
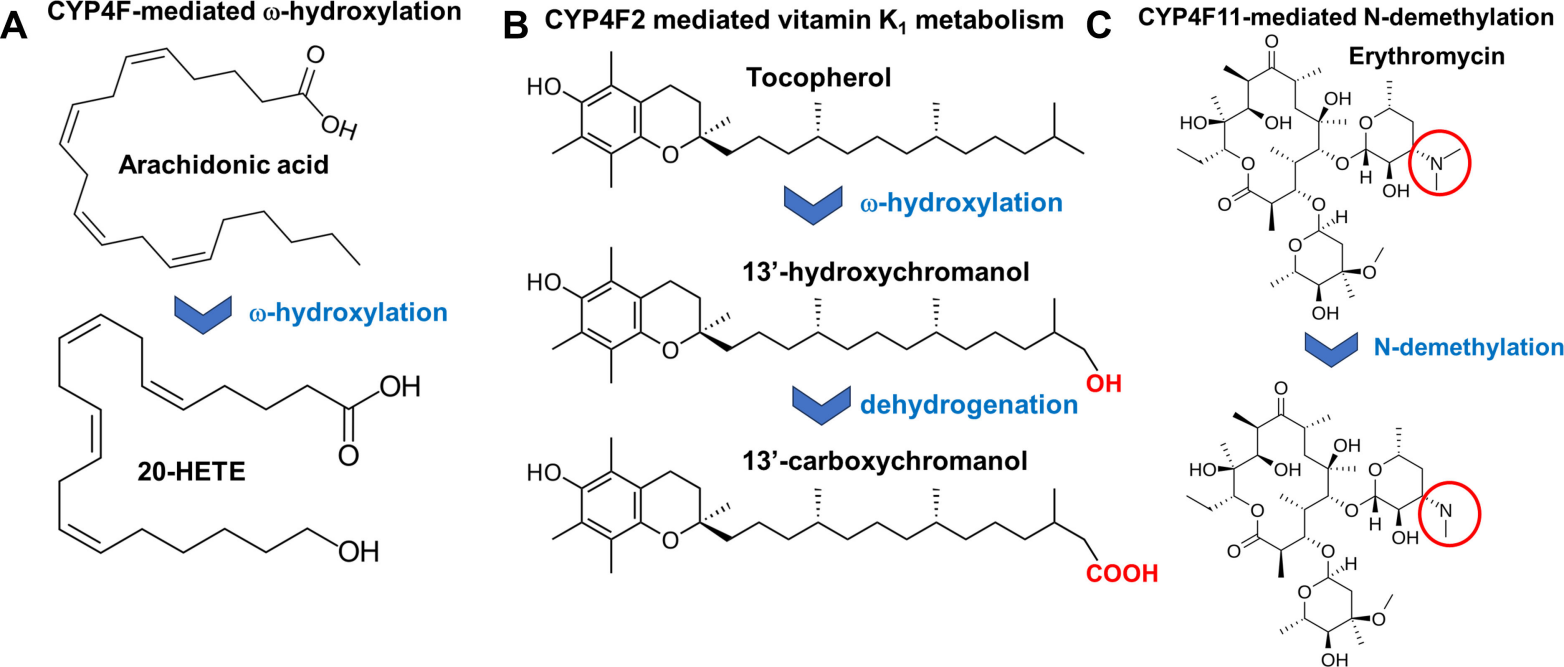
The ω-hydroxylation of arachidonic acid to 20-HETE is the major signature reaction of most CYP4F enzymes (A). The isoform CYP4F2 is the only one able to perform an ω-hydroxylation and then subsequent dehydrogenation of vitamin E (tocopherol) to the biologically significant metabolite 13′-carboxychromanol (B). CYP4F11 stands out from other CYP4F isoforms due to its efficient metabolism of drugs such as the macrolide antibiotic erythromycin though an unusual N-demethylation, a function which CYP4F11 exhibits in addition to fatty acid ω-hydroxylation (C).

CYP4F2 primarily catalyses the oxidation of the terminal carbon of long- and very long-chain fatty acids but is also known for metabolizing vitamins [26,27]. The CYP4F2 isoform was first described by Kikuta and collaborators in 1994 as a novel enzyme able to hydroxylate leukotriene B4 (LTB4) [28]. CYP4F2 had a *K*_m_ for LTB4 of 44.8 µM and was expressed exclusively in the liver. The conversion of arachidonic acid to the lipid mediator 20-HETE as a regulator of blood pressure and angiogenesis by CYP4F2 was later confirmed with a *K*_m_ of 24 µM in liver microsomes [29]. This work was the first description of CYP4F2 as a major ω-hydroxylase in the production of 20-HETE (figure 2A). More information about the CYP4F2 gene structure, protein expression and substrate scope was elucidated in the following years. The gene encoding CYP4F2 is located on chromosome 19. The substrate scope includes LTB4 with a *K*_m_ of 60 µM, 6-trans-LTB4 with a *K*_m_ of 55.6 µM, lipoxin A with a *K*_m_ of 58.2 µM, 8-HETE with a *K*_m_ of 19 µM, 12-HETE with a *K*_m_ of 42.3 µM and 12-hydroxysterate with a *K*_m_ of 75.2 µM [30]. Even though there are substantial differences between reported *K*_m_ values for the same substrate due to different types of *in vitro* systems, this work shows that CYP4F2 has a broad substrate range among fatty acids and eicosanoids. Besides endogenous substrates, CYP4F2 can metabolize a diverse spectrum of drugs spanning from chemo-therapeutic drugs like imatinib, the anti-parasite drug pafuramidine to sphingosine l-phosphate receptor modulator fingolimod [31–33]. These drugs do not have many structural similarities. Thus, CYP4F2 is a versatile protein capable of metabolizing a diverse set of xenobiotics.

In 2019, it was demonstrated that hepatic CYP4F2 plays a crucial role in the metabolism of benzalkonium chlorides (BACs) [34]. BACs are widely used antimicrobial agents found in disinfectants, medical products and the food processing industry. The use of BACs was particularly widespread during the COVID pandemic. Their toxicity has been linked to an accumulation in the kidney. Interestingly, no significant BAC accumulation has been observed in liver, likely due to CYP4F2-mediated metabolism of BACs via an ω-hydroxylation of the alkaloid chain [35]. The hepatic metabolism may help prevent BAC-induced liver toxicity and provides valuable insights into potential strategies for mitigating BAC-related adverse effects.

#### 2.2. Gene regulation

In terms of the induction of CYP4F2 expression, it was first reported in 2000 that the CYP4F2 gene is induced by retinoic acid and fatty acids [36]. CYP4F2 is regulated by retinoic acid, with the retinoid X receptor (RXRα) stimulating gene expression while the retinoic acid receptors (RARα) repress the expression. Hsu and collaborators described for the first time that CYP4F2 gene expression is induced by lovastatin and sterol regulatory proteins in HepG2 cells [37]. Statins, such as lovastatin, are used for treatment of high cholesterol in serum. They act as inhibitors of the enzyme 3-hydroxy-3-methylglutaryl-coenzyme A (HMG-CoA) reductase, involved in cholesterol synthesis. The CYP4F2 gene induction is mediated by the sterol regulatory element-binding proteins (SREBPs). The SREBPs are DNA-binding proteins with leucine zipper structure. They are responsible for regulating genes involved in cholesterol and fatty acid synthesis. The authors also report that lovastatin increases CYP4F2 mRNA by threefold in HepG2 cells and by fivefold in hepatocytes. They hypothesize that CYP4F2 gene activation by SREBP blocks the high flux of triglycerides and fatty acids due to an increased LDL import to the liver cells. Additional studies are required to further confirm this mechanism. It was then discovered that CYP4F2 gene expression is induced by activators of AMP-activated protein kinase (AMPK) [38]. AMPK is important for the regulation of fatty acid oxidation. The indirect activator 5-aminoimidazole-4-carboxamide-1-β -D-ribofuranoside (AICAR) leads to a 2.5-fold increase in CYP4F2 mRNA expression. This increase was not observed with other CYP4F enzymes evaluated in HepG2 cells. The use of a specific inhibitor of AMPK led to a reduction in CYP4F2 expression. Conversely, indirect activators of AMPK, genistein and resveratrol, lead to an increase of CYP4F2 mRNA. AMPK is activated when AMP/ATP ratios in cells are high. After AMPK phosphorylation, transcription factors can activate the CYP4F2 gene. This CYP4F2 activation is thought to avoid an accumulation of fatty acids to toxic levels. Additional studies to endorse this mechanism are still lacking to date.

#### 2.3. The role of CYP4F2 in warfarin dose response

A CYP4F2 gene polymorphism was first described in a genome association study for warfarin administration. Warfarin is a well-established anticoagulant used for thrombosis treatment. Cooper and collaborators were the first group to genotype the CYP4F2 rs2108622 polymorphism as part of a genome-wide association study conducted with 181 patients receiving warfarin, which leads to the amino acid exchange V433M [39]. In this study, the single-nucleotide polymorphism (SNP) rs2108622 was reported, however, with a small size effect (1–2%) variation in warfarin dose. This size did not represent a statistical significance in warfarin impairment dosage, but the significance of this polymorphism was confirmed in the same year.

Another study confirmed that CYP4F2 rs2108622 V433M variant is related to a difference in the required warfarin dose for drug usage [40]. The differences in warfarin dosage were approximately 1 mg per day between CC and TT alleles. The authors concluded that this genetic variation was associated with clinical effect on warfarin dosage. In addition, it was discovered that the variant has an altered enzyme activity. The SNP individual allele variant presented a 60% reduction in 20-HETE production compared to the wild-type (WT). Still, the role of CYP4F2 in vitamin K metabolism and its association with an altered warfarin dose response was inconclusive.

In 2009, the function of CYP4F2 in vitamin K metabolism was first reported [41]. It was revealed that CYP4F2 is a vitamin K_1_ (VK1) oxidase presumably performing an ω-hydroxylation of the phytyl side chain rather than the menadione head group. It was also established that interindividual variability in warfarin dosage is correlated to genetic polymorphisms in VKORC1 and CYP2C9, the genes responsible for vitamin K_1_ epoxide reduction (VKORC1) and (*S*)*-*warfarin metabolism, respectively. It was also confirmed that the CYP4F2 variation V433M is associated with warfarin dose and speculatively linked to modified VK_1_ metabolism. A screen of all available P450 supersomes showed that only CYP4F2 was capable of metabolizing VK1 to hydroxyvitamin K_1_ with a *K*_m_ of around 10 µM. Human liver microsomes genotyped for the V433M variant showed reduced VK1 oxidation and lower CYP4F2 protein concentrations. A comprehensive analysis of the V433M variant on warfarin dosage requirements was published in 2012 [42].

**Figure 3.**
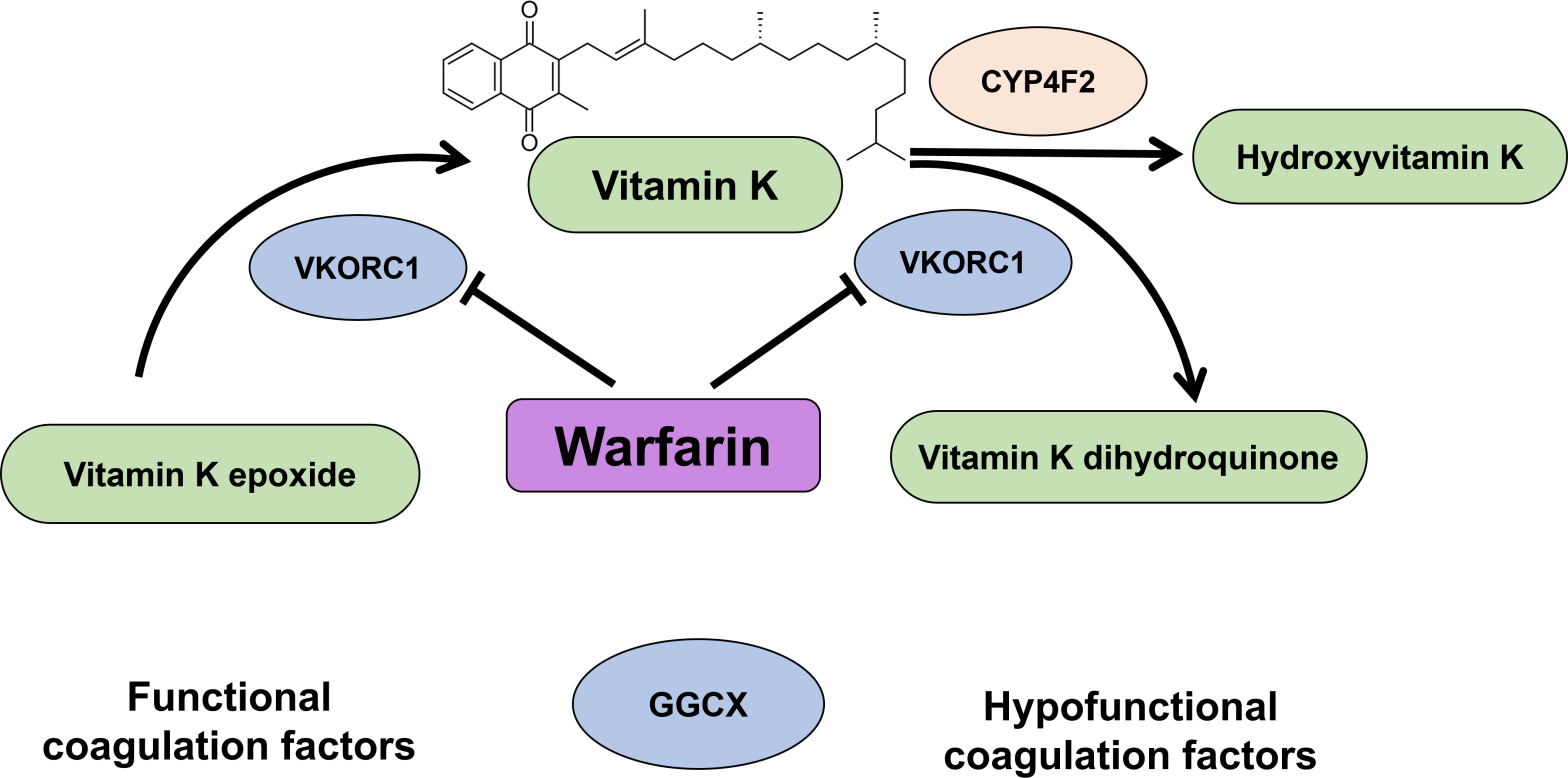
The enzyme vitamin K_1_ epoxide reductase (VKORC1) activates vitamin K_1_ to vitamin K_1_ dihydroquinone which is a cofactor for the enzyme γ-glutamyl carboxylase, the enzyme responsible for activating blood factor proteins which initiates the blood coagulation cascade. This step converts vitamin K_1_ dihydroquinone to vitamin K_1_ epoxide. VKORC1 is thought to catalyse at least one step in the regeneration of vitamin K_1_ from vitamin K_1_ epoxide. Warfarin acts on VORC1 and prevents blood clotting events as a blood thinner. CYP4F2 influences this cycle by removing vitamin K_1_ through hydroxylation to hydroxyvitamin K_1_, and, thus, indirectly influences blood clotting. (Figure adapted from [43].)

Consequently, the role of CYP4F2 in vitamin K_1_ (VK1) metabolism and the interplay with VKORC1 was revealed [41]. CYP4F2 converts VK1 into hydroxyvitamin K_1_, but VK1 is also converted to its active form vitamin K_1_ dihydroquinone (VKH2) by the reductase VKORC1. VKH2 is a crucial cofactor for γ-glutamyl carboxylase (GGCX) which carboxylates specific glutamate residues crucial for activation of blood factor proteins. After this activation, the blood coagulation cascade is initiated. Thus, vitamin K_1_ is crucial for blood clotting and bone health. In concomitance to this activation, VKH2 is oxidized to 2*S*,3*R*(+)vitamin K_1_ epoxide (KO). Warfarin acts specifically on the VKORC1 reductase enzyme. VKORC1 reductase catalyses at least the first of the reductions and regenerates VK1 from the KO form. CYP4F2 affects warfarin dose response through the removal of VK1 from the cycle (figure 3). The CYP4F2 polymorphism impairs protein function and leads to an accumulation of VK1. VK1 is then activated by VKORC1 resulting in a higher occurrence of blood clotting events which ultimately requires a higher warfarin dosage. CYP2C9 is the enzyme responsible for warfarin clearance in the liver. Thus, genetic variations of these three enzymes, CYP4F2, VKORC1 and CYP2C9, have to be accounted for when adjusting warfarin dosage. More studies on genetic variants and interplay of these three enzymes can be found in comprehensive studies reported in [44–46].

Edson and collaborators reported that human CYP4F2 and CYP4F11 were able to ω-hydroxylase the menaquinone form of vitamin K (MK4), also known as vitamin K_2_ [13]. MK4 has multiple biological roles, such as the involvement in mitochondrial electron transport, as a ligand of NRs, and as a pharmacotherapeutic used in the treatment of osteoporosis. Both CYP4F2 and CYP4F11 are efficient in the catalysis of MK4. CYP4F2 has the capacity to metabolize MK4 in a sequential reaction to an ω-acid form, which was not observed for CYP4F11. Thus, CYP4F2 is assumed to contribute to MK4 catabolism, in particular of dietary MK4.

A recent study evaluated the impact of the V433M SNP from a structural perspective [47]. Molecular dynamics (MD) simulations assessed the impact of V433M mutation on CYP4F2 structure, stability and dynamics and found that the V433M variant affected protein dynamics and reduced overall protein stability. The V433M variant changed structural conformation and flexibility of CYP4F2 and exhibited a decreased binding to VK1 potentially causing a reduced activity of the variant compared to WT CYP4F2. However, a study published in 2016 reported no change in catalytic activity but drastically decreased protein levels potentially due to a decrease in stability which underlines the need to relate catalytic function to protein expression levels [48].

#### 2.4. CYP4F2-mediated metabolism of vitamin E

CYP4F2 is also involved in the metabolism of vitamin E, also known as tocopherol. Sontag and Parker reported for the first time that only CYP4F2 exhibits tocopherol-ω-hydroxylase activity [49] (figure 2B). Recombinant CYP4F2 was capable of metabolizing γ-tocopherol and α-tocopherol to 13′-OH and 13′-COOH, respectively. While α-tocopherol is considered the most active and biologically significant form of vitamin E, γ-tocopherol is mostly a dietary component and not used to treat vitamin E deficiency. However, both forms have been reported to exhibit antioxidant properties, protecting cells from damage associated with oxidative stress. Interestingly, CYP4F2 displays a higher enzymatic activity towards plant-derived tocotrienols compared to tocopherols, indicating a preference of CYP4F2 for tocotrienol as substrates [49]. Only very recently, it was reported that the 13′-COOH metabolites exhibit anti-inflammatory and anti-cancerogenic effects which might contribute to the overall beneficial and disease-preventing effects of vitamin E.

Bardowell and collaborators reported *in vivo* studies with the CYP4F2 mouse orthologue CYP4F14 [50]. A genetic knockout of CYP4F14 led to a decrease of urinary and fecal excretion of vitamin E metabolites and an increase in tissue concentration of tocopherols compared to WT mice. The authors estimated that CYP4F2 accounts for 70–80% whole body vitamin E metabolites [51]. More work is needed to elucidate the effects of CYP4F2-mediated metabolism of vitamin E derivatives and the potential therapeutic application.

#### 2.5. The role of CYP4F2 in hypertension, cardiovascular disease and ischemic stroke

The lipid mediator 20-HETE, which is the major product of CYP4F2 metabolism of arachidonic acid, has vasoconstrictive properties and, thus, can increase blood pressure. In the kidney, however, 20-HETE also exhibits natriuretic effects which lead to a local decrease in blood pressure [52,53]. As previously mentioned, the CYP4F2 rs2108622 SNP c1347G>A leads to the amino acid exchange V433M which is associated with a reduced metabolism of amino acids *in vitro* [54]. Curiously, Ward and collaborators reported that the variant V433M increased the urinary excretion of 20-HETE which was associated with high systemic blood pressure, although the variant has reportedly decreased enzyme activity [55]. The authors concluded that the effect of the enzyme variant might be indirect. It may lead to the upregulation of other 20-HETE producing P450 enzymes which then overcompensate reduced CYP4F2 function. Since then, these findings have been confirmed in other population cohort studies [56,57]. Besides that, V433M and additional CYP4F2 alleles which do not lead to amino acid exchanges were associated with ischemic stroke and cerebral infarction in Swedish males, the Han Chinese population and a Japanese cohort [57–60].

Recently, it was shown that specific N-acyl amino acids are associated with cardiometabolic disease endpoints. CYP4F2-mediated hydroxylation of N-oleoyl-leucine and N-oleoyl-phenylalanine results in metabolic diversification and production of many previously unknown lipid molecules [61]. These functionalized lipids present characteristics resembling ω-hydroxy fatty acids, which could potentially act as signalling molecules. This work provides a framework for understanding the regulation and association of N-acyl amino acids in cardiac disease and how this is expanded through CYP4F-mediated ω-hydroxylation. However, the exact role of CYP4F2 has to be further elucidated in the future.

#### 2.6. Implications of the role of CYP4F2 in cancer

The crucial role of fatty acid and eicosanoid metabolism in cancer has long been known and offers new strategies for cancer therapies. 20-HETE has pro-proliferative properties and a positive impact on metastasis. On top, 20-HETE promotes angiogenesis, providing a tumour with new blood vessels for its growth. However, only little is known about CYP4F isoform-specific roles in various cancer types and if their role expands beyond simply producing 20-HETE.

A study focusing on P450 family 4 gene expression in human hepatocellular carcinoma revealed that CYP4F2 expression is a favourable prognostic factor suggesting a potential predictive diagnostic role [62]. Other findings suggest that CYP4F2 is downregulated in hepatocellular carcinoma and that low expression of CYP4F2 is correlated with reduced patient survival [63]. Chen *et al.* investigated the interconnection between CYP4F2 and immune evasion in non-small cell lung cancer (NSCLC) [9]. They confirmed that 20-HETE drives immune evasion in a CD8+T cell dependent manner and induces the expression of the programmed cell death ligand-1 (PD-L1), an immune checkpoint. CYP4F2-mediated 20-HETE production also triggered the production of interleukin-6 (IL-6) and TGF-β in cancer-associated fibroblasts (CAF) leading to immune evasion through the GPR75-STAT3-c-Jun axis. In addition, NSCLC patients with high levels of CYP4F2 were more resistant to anti-PD-1 immunotherapy. General unselective CYP4F inhibition, not of CYP4F2 specifically, improves the efficacy of anti-PD-1 therapy in tumours resistant to immunotherapy. The authors claim that the inhibition of CYP4F2 enzyme is a potential therapeutic approach for immunotherapy-resistant lung cancer. This study presents the first link between CYP4F2 and immune evasion in cancer, but future work is necessary to reveal the exact CYP4F2 function and how this can be extended to the other CYP4F isoforms.

Recently, a link between CYP4F2 and CYP4F11 expression and the progression of estrogen receptor-positive (ER+) breast cancer was established [64]. The study evaluated both overexpression and knockout of CYP4F2 and CYP4F11 and their effects on cell proliferation, apoptosis and tumour growth. An overexpression of both CYP4F isoforms was induced by estrogen which stimulated cancer cell proliferation. Besides that, an overexpression led to resistance to apoptosis due to 20-HETE production, which activated the antiapoptotic protein Bcl-2. Both enzymes were considered potential markers for breast cancer pathology. In summary, their inhibition could provide a new treatment option for ER+ breast cancer.

Recent studies show that CYP4F2 expression is related to hepatocellular carcinoma, NSCLC and breast cancer. However, the respective role of CYP4F2 seems to be different in different cancer types. Thus, future studies need to address the cancer-specific action of CYP4F2 for the future development of targeted therapies.

### 3. The CYP4F3 splice variants—unique among human P450 enzymes

#### 3.1. Discovery and substrate scope

**Figure 4.**
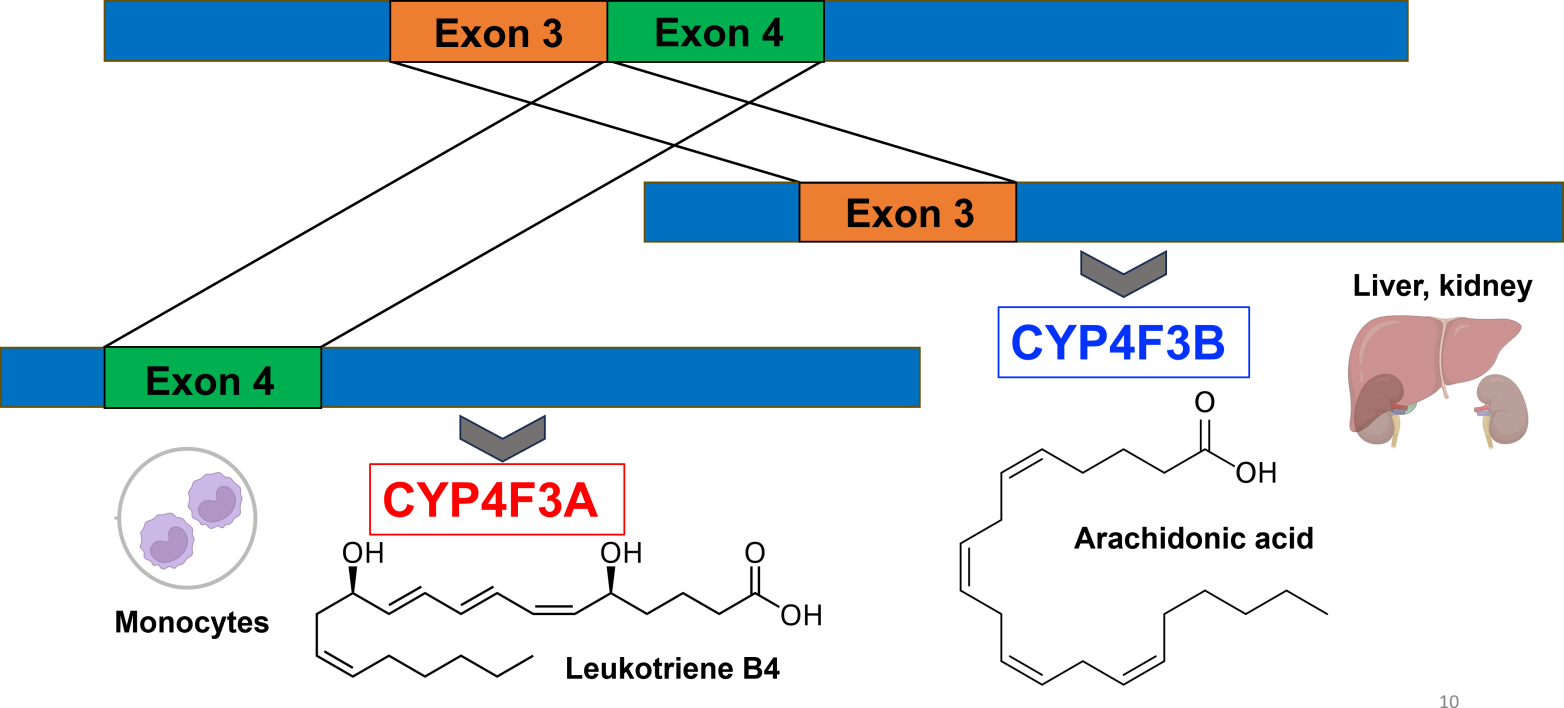
Both CYP4F3 isoforms are encoded by the same gene, and their expression is controlled by alternate splicing events. Monocytes exclusively express the CYP4F3A variant through insertion of exon 4. The resulting protein is contributing to the anti-inflammatory response by inactivation of leukotriene B4 though ω-hydroxylation. In liver and kidney, exon 3 is inserted resulting in the variant CYP4F3B which is a major producer of 20-HETE through ω-hydroxylation of arachidonic acid.

The CYP4F3 proteins play a crucial role in the inflammatory response in the human body. Studies in the 1980s described the ω-hydroxylation of leukotriene B4 (LTB4) as a mechanism for inflammation resolution [65–67]. In the 1990s, the CYP4F3 gene, which is located on chromosome 19, was cloned, and its function was further elucidated. Besides LTB4, CYP4F3 also catalyses the ω-hydroxylation of long-chain polyunsaturated fatty acids (PUFAs), long-chain fatty acid epoxides and arachidonic acid [27,28,68]. These early studies suggested that CYP4F3 was a single enzyme. However, shortly after, Christmas and collaborators established that two CYP4F3 isoforms exist, CYP4F3A and CYP4F3B, which are derived from a unique alternative splicing event from the same gene locus [11]. Each enzyme has a specialized function for different substrates in a tissue-dependent manner. The exon splicing is an exceptional event among human P450 enzymes. The CYP4F3A mRNA includes exon 4, while CYP4F3B mRNA incorporates exon 3 (figure 4). CYP4F3A performs the ω-hydroxylation of LTB4 which results in an anti-inflammatory response, while CYP4F3B acts as an ω-hydroxylase of arachidonic acid producing 20-HETE (figure 2A) [11,69]. The CYP4F3 isoforms are expressed in different tissues underlining their distinct biological functions. CYP4F3A is exclusively produced in monocytes while CYP4F3B is produced in liver and kidney. CYP4F3A has a higher affinity for LTB4 (*K*_m_ = 0.68 µM) compared to CYP4F3B (*K*_m_ = 20.6 µM). Conversely, CYP4F3B has a higher affinity for arachidonic acid (*K*_m_ = 22 µM) compared to CYP4F3A (*K*_m_ = 185.6 µM) [11,69]. CYP4F3A and CYP4F3B share 93% amino acid sequence identity. The only difference between these proteins is restricted to a specific amino acid stretch spanning residues 67–114 which is directly affected by the alternate splicing event. This region shares only 27% sequence identity. As discussed previously, the exon swap region seems to be determinant for substrate access and recognition. The splice variants CYP4F3A and CYP4F3B provide evidence for the impact of this amino acid region on substrate preferences and distinct physiological functions.

The substrate scope of CYP4F3B was further elucidated with a focus on PUFAs [27]. CYP4F3B was the most efficient enzyme for the ω-hydroxylation of omega-3 fatty acids. CYP4F3B is also responsible for the ω-hydroxylation of eicosapentaenoic acid (EPA) and docosahexaenoic acid (DHA).

#### 3.2. CYP4F3 gene regulation

CYP4F3A gene regulation was first elucidated by Christmas and collaborators [70]. The authors reported that CYP4F3A expression is activated in myeloid tissue by the transcription factor ZEB-2, a zinc finger transcription factor. ZEB-2 was able to interact with the C-terminal binding protein and Smads proteins, which act as signal transducers. Smads are related to TGF-β signalling and thus control the inflammatory response. Hence, the mechanism is integrated with immune system regulation and inflammation occurrence. The CYP4F3A gene and enzyme activity are also induced by all-*trans* retinoic acid (ATRA), which leads to LTB4 hydroxylation and inflammation resolution [71]. The CYP4F3A protein induction was evaluated in white blood cells of patients exposed to benzene. The presence of phenol, a metabolite of benzene, leads to an increase of CYP4F3A mRNA and protein in HL-60 cells. However, this effect was not observed with benzene alone. Recently, it was reported that CYP4F3 gene overexpression could upregulate NRF2 (nuclear factor erythroid 2-related factor 2). NRF2 is related to the oxidative stress response [72]. However, the specific CYP4F3 splice variant was not determined in this study.

CYP4F3B gene expression is stimulated by prostaglandin A_1_ (PGA_1_) in human hepatocytes (HepaRG) [73]. The expression of CYP4F3B mRNA was induced in a concentration-dependent manner leading to the production of 20-HETE. In 2012, Plée-Gautier and collaborators established that CYP4F3B gene expression is regulated by lovastatin, a cholesterol-lowering drug, in HepaRG cells [74]. Lovastatin induces CYP4F3B mRNA production and protein expression with an increase in 20-HETE production. The authors also showed that the pregnane X receptor (PXR) mediates CYP4F3 induction by lovastatin and concluded that CYP4F3B products could modulate lipid metabolism in response to lovastatin, similar to CYP4F2.

#### 3.3. The CYP4F3 splice variants in human disease

Multiple studies report a correlation between the CYP4F3 splice variants and the development of cancer, immune disorders, and heart disease. CYP4F3 was considered a candidate gene in SNP studies for bronchial asthma [75]. CYP4F3A is associated with inflammatory bowel diseases and Crohn’s disease [76,77]. A study reported that children with a higher dietary ratio of ω-6 to ω-3 PUFAs were more susceptible to Crohn’s disease if they carried variants of the CYP4F3 gene [78]. In addition, the CYP4F3 rs3794987 G allele was higher in women with gestational preeclampsia in a Turkish cohort [79].

Yin and collaborators studied the potential role of the CYP4F3 gene locus in lung cancer [80]. The authors reported a meta-analysis of published datasets of six genome-wide association studies (GWASs) from the Transdisciplinary Research in Cancer of the Lung (TRICL) consortium. The databases present cases with lung cancer and cancer-free controls. Here, the CYP4F3 SNP rs4646904 G>A is significantly associated with a higher lung cancer risk. The authors discuss that alternative splicing might decrease CYP4F3A expression, resulting in increased levels of LTB4 while elevating the expression of CYP4F3B and, thus. the production of 20-HETE. The same SNP rs4646904 was associated with ulcerative colitis in a different genome study [81].

Smeets *et al.* reported a point mutation that results in high levels of LTB4 in plasma and a reduced immune response [82]. This point mutation leads to the amino acid exchange L375V in the catalytic domain of CYP4F3 and impairs the enzymatic activity through destabilization of an α-helix important for electron transfer.

Recent studies suggest a potential role of CYP4F3 in the progression of colorectal cancer [72]. The CYP4F3 gene was upregulated and associated with poor survival. The authors describe that upregulation of CYP4F3 leads to an increased cell proliferation and migration. Besides that, overexpression of CYP4F3 resulted in an upregulation of NRF2 which inhibits cellular ferroptosis. Thus, CYP4F3 could be a potential therapeutic target for colorectal cancer. However, the cellular mechanism remains to be understood. While the correlation between CYP4F11 and NRF2 was previously described, the relationship between CYP4F3 and NRF2 is not clear to date. As we elaborate later in this paper, the expression of the isoform CYP4F11 is regulated by NRFR2. Thus, more studies are needed to elucidate if CYP4F3 and NRF2 are intertwined in a similar fashion. In addition, there are limited studies for the gain and loss of function of CYP4F3 in different cancers distinguishing the isoforms CYP4F3A and CYP4F3B. Since CYP4F3A acts in inflammation resolution and CYP4F3B is responsible for 20-HETE production, they could potentially have distinct functions in disease progression.

Johnson and co-workers provided a review of the CYP4F family in inflammation and cancer and highlighted the lack of comprehensive research in 2015, which is already 10 years ago [83]. This emphasizes the urgent need for in-depth studies on this matter.

### 4. CYP4F11—the unicorn among the CYP4F isoforms

#### 4.1. Discovery and substrate scope

CYP4F11 plays a dual role in metabolizing both xenobiotic compounds and fatty acids. It was first identified in human liver in 2000 and further characterized when recombinant protein could be expressed in yeast cells [10,14]. CYP4F11, like other P450 ω-hydroxylases, was initially believed to primarily hydroxylate endogenous fatty acids, particularly eicosanoids. However, these early studies also revealed that CYP4F11 also acts as a drug-metabolizing enzyme. In 2010, recombinant human CYP4F11 was successfully expressed and purified using an *Escherichia coli* expression system, demonstrating its catalytic activity across a panel of fatty acids in human liver extracts [84]. Since then, research on CYP4F11 has expanded significantly, focusing on its enzymatic function, gene regulation and clinical implications.

Like other ω-hydroxylases, CYP4F11 is located on chromosome 19, approximately 16 kb upstream of the CYP4F2 gene. Notably, the CYP4F11 transcript comprises 12 exons, in contrast to other CYP4F isoforms, which typically contain 13 exons. CYP4F11 shows activity towards both endogenous and exogenous compounds [14]. CYP4F11 has has a broad substrate scope and is capable of oxidizing fatty acids and eicosanoids [10]. The catalytic activity was evaluated among a variety of eicosanoids and fatty acids, including LTB4, arachidonic acid (figure 2A), lipoxin A4 (LXA4) and B4 (LXB4), 5-, 8-, 12-HETE, and prostaglandin A1 and E1. However, CYP4F11 shows a generally lower catalytic activity towards eicosanoids than CYP4F3A and no activity towards 5- and 12-HETE. It has also been shown that CYP4F11 metabolizes 15-HETE. Most strikingly, this metabolic function is related to malaria-induced immune reactions [85]. In addition, CYP4F11 expressed in insect cells exhibits ω-hydroxylase activity towards 3-hydroxyl fatty acid and is the only CYP4F enzyme capable of performing this reaction. Here, CYP4F11 function is assumed to serve as backdoor pathway for fatty acid clearance in β-oxidation disorders [15]. In 2010, it was discovered that more fatty acids are substrates of CYP4F11 such as palmitic acid (C16:0), oleic acid (C18:1) and DHA (C22:6), which is the most abundant fatty acid in the brain [84]. Notably, CYP4F11 is more broadly expressed among different tissues other than liver and kidney with a significant expression in the brain.

CYP4F11, along with CYP4F2, is a key player in vitamin K_2_ functionalization [13]. The previously mentioned vitamin K_2_ MK4 acts as a ligand of steroid and xenobiotic receptor (SXR), regulating the expression of genes involved in transport and metabolism of both endogenous and xenobiotic compounds. CYP4F11 is capable of oxidizing MK4 and phylloquinone (PK), but with lower efficiency compared to CYP4F2. Furthermore, while CYP4F2 generates an ω-carboxylated metabolite, this product was not detected in CYP4F11 metabolism assays. Thus, compared to CYP4F2, CYP4F11 appears to be a less dominant contributor to the initial steps of MK4 metabolism. MK4 has been approved in Japan for the prevention and treatment of osteoporosis in women, while PK is routinely administered to newborns in the USA to prevent vitamin K deficiency bleeding. CYP4F11 has at least one common genetic variant (D446N) arising from the SNP rs1060463. This SNP has a minor allele frequency of approximately 40% in the Caucasian population. However, no significant difference in CYP4F11 protein level between the WT and the mutated enzyme could be detected in human liver microsomes. Moreover, MK4 ω-hydroxylation remains unaffected by the CYP4F11 variant [13].

In summary, CYP4F11 contributes to MK4 metabolism but to a far lesser extent than CYP4F2.

#### 4.2. CYP4F11-mediated metabolism of xenobiotics

CYP4F11 stands out from other CYP4F isoforms due to its ability to efficiently metabolize drugs. It performs an N-demethylation of the antibiotic erythromycin (figure 2C), the amphetamine benzphetamine, the opioid ethylmorphine, the tricyclic antidepressant imipramine, the bronchodilator theophylline and the calcium channel blocker verapamil [10]. The fact that CYP4F11 is expressed in the brain is particularly interesting when it comes to the metabolism of drugs used for the treatment of depression and other psychiatric disorders.

CYP4F11 expressed in yeast microsomes converts erythromycin with a *K*_m_ of 125 µM and V_max_ of 830 pmol min^−1^ nmol^−1^, which makes it the most efficient substrate of CYP4F11 with a catalytic activity comparable to that of CYP3A4. Collectively, CYP4F11 is far more efficient in metabolizing drugs compared to other CYP4F enzymes and should not be neglected in pharmacokinetic studies [86]. Future studies are necessary to understand the full capacity of CYP4F11 to metabolize drugs and to elucidate the full substrate scope.

Recently, the high abundance of CYP4F11 in lung cancer tumours made it an attractive tool for prodrug activation targeting the stearoyl CoA desaturase (SCD) [87,88]. Usually, inhibiting SCD leads to serious side effects due to its broad expression. CYP4F11 is able to activate prodrugs by performing an O-demethylation of benzothiazole and oxalamide compounds. The activated compound then leads to an irreversible inhibition of SCD. Since SCD has been identified as a powerful therapeutic target in cancer, the CYP4F11-mediated prodrug activation provides a strategy to inhibit SCD locally with reduced side effects.

These findings highlight the dual role of CYP4F11 in both endobiotic and xenobiotic metabolism, which sets CYP4F11 apart from other members of the CYP4Fs family. Notably, its active site seems to be highly flexible and able to accommodate large substrates such as erythromycin while maintaining the difficult ω-hydroxylation function. This versatility invites further investigation into the enzyme’s structural properties.

#### 4.3. CYP4F11 gene regulation and its implication in cancer

CYP4F11 mRNA is expressed mainly in the human liver, followed by the kidney, heart, skeletal muscle, and brain [10]. In 2010, it was shown that the CYP4F11 gene is positively regulated by multiple signalling pathways in HaCaT keratinocytes, including the retinoid X receptor (RXR) and the c-Jun N-terminal kinase JNK signalling pathways [89]. Retinoic acids, derived from vitamin A (retinol), regulate gene expression through nuclear receptors (NRs), retinoic acid receptors (RARs) and the RXRs [90]. Notably, the Strobel team found that retinoic acids suppress CYP4F11 expression in HaCaT cells. This suppression occurs through the interaction between its receptors and the activator protein 1 (AP-1) complex, a key transcript or factor involved in inflammatory and stress responses. Conversely, proinflammatory cytokines, such as tumour necrosis factor alpha (TNF-α) and interleukin 1 beta (IL-1β), induce CYP4F11 transcription by activating the AP-1 complex. This induction is dependent on the JNK signalling pathway, as demonstrated by the ability of the JNK-specific inhibitor SP600125 to block cytokine-mediated CYP4F11 upregulation. Furthermore, preincubation with retinoic acids prevents TNF-α from inducing CYP4F11 transcription, suggesting a functional interplay between RXR signalling and the JNK/AP-1 pathway. These findings highlight a dynamic regulatory mechanism in keratinocytes, where retinoic acid signalling modulates inflammatory gene expression of CYP4F11 by interfering with AP-1 activity. The biological significance of this finding is yet to be determined but implicates a role of CYP4F11 during inflammation.

**Figure 5.**
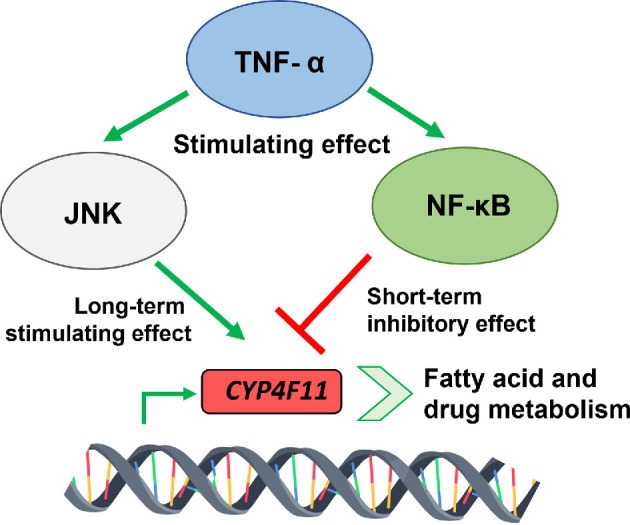
CYP4F11 expression during inflammation is an intricate interplay between the two inflammation master regulators JNK (c-Jun N-terminal kinase) and NF-κB (nuclear factor kappa-light-chain-enhancer of activated B cells). While TNF-α (tumour necrosis factor alpha) stimulates both regulators, JNK enhances CYP4F11 expression while NF-κB represses it. Curiously, the inhibitory effect of NF-κB is only short term eventually leading to a gene expression enhancement by JNK long term [90,91].

In 2012, the Strobel group further investigated the regulatory complexity of NF-κB-mediated CYP4F11 regulation during inflammation [91]. In the presence of TNF-α and the specific NF-κB translocation inhibitor *N*-[3,5-bis(trifluoromethyl)phenyl]-5-chloro-2-hydroxybenzamide (IMD-0354), the CYP4F11 expression was significantly higher than with TNF-α alone. Moreover, NF-κB activation via mitogen-activate protein kinase (MAPK) overexpression inhibited CYP4F11 promoter function. This inhibition was reversed when p65, a key NF-κB subunit, was knocked down in the presence of TNF-α, further confirming the suppressive effect of NF-κB on CYP4F11 expression. Since TNF-α activates both JNK and NF-κB pathways, the Strobel group proposed that these pathways regulate opposing gene expression outcomes (figure 5) [91]. Prolonged TNF-α stimulation leads to sustained JNK activation, which enhances CYP4F11 expression, whereas NF-κB activation suppresses CYP4F11 expression and only acts short term. These findings underscore the intricate balance between pro-inflammatory and regulatory signalling pathways in controlling CYP4F11 gene expression and emphasize the complex interplay between JNK and NF-κB during inflammation. Further evidence that CYP4F11 might be involved in the anti-inflammatory response was provided in 2021 in a study which was focused on the liver X receptor (LXR) [92].

In 2017, it was reported that CYP4F11 is positively regulated by NRF2 in NSCLC [93]. NRF2 is a transcription factor critical for the cellular antioxidant response and is negatively regulated by KEAP1 (Kelch-like ECH-associated protein 1). Under normal physiological conditions, KEAP1 binds to NRF2, leading to its ubiquitination and subsequent degradation. However, in cancer, KEAP1 is often mutated which impairs its interaction with NRF2. This results in NRF2 stabilization, nuclear translocation and pro-proliferative gene expression. Importantly, the dysregulation of NRF2 triggered by mutated KEAP1 leads to a drastic upregulation of CYP4F11. A genetic knockout of CYP4F11 in lung cancer cell lines attenuated the cancer cell colony formation suggesting that CYP4F11 plays a role in NRF2-dependent lung cancer progression. In 2024, our laboratory provided further evidence supporting the pro-proliferative role of CYP4F11 [18]. We demonstrated its ability to promote both proliferation and migration of lung cancer cells which is accompanied by a reduced 20-HETE production. This further emphasizes the potential for a new therapeutic option for lung cancer treatment.

As already described for CYP4F2, both CYP4F2 and CYP4F11 are associated with ER+ breast cancer and are regulated by estrogen. As observed for CYP4F2, the genetic ablation of CYP4F11 led to a decreased proliferation of a breast cancer cell line with reduced reduction of 20-HETE [64].

### 5. CYP4F22—a key player in the skin barrier

CYP4F22 is the most distinct among the CYP4F isoforms due to its early evolutionary divergence from other CYP4F family members. Long considered an orphan P450 isoform, its biological function has only recently been elucidated. Before this, CYP4F22 had been known since 2006 to be associated with the skin disease lamellar ichthyosis, which ultimately led to the quest to unravel CYP4F22 function [94]. Lamellar ichthyosis is a genetic disorder that leads to the desquamation of the skin due to a dysfunctional stratum corneum.

**Figure 6.**
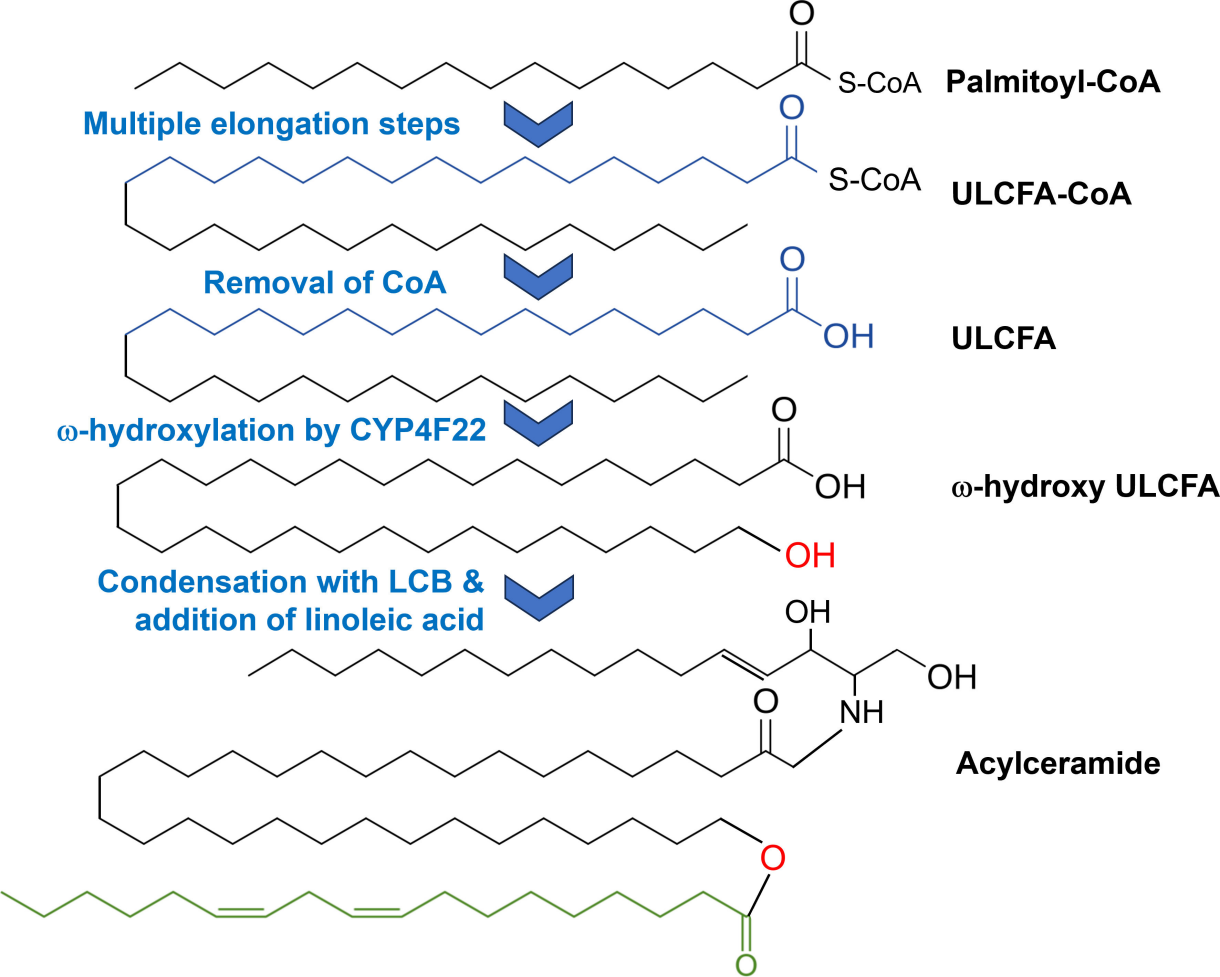
Acylceramide biosynthesis starts with a fatty acid CoA (here, palmitoyl-CoA) which is elongated through multiple steps to an ultra-long-chain fatty acid (ULCFA). After removal of the CoA group, the ULCFA is subsequently ω-hydroxylated by CYP4F22. Further steps include the condensation with a long-chain base (LCB) and the addition of another fatty acid (here, linoleic acid) to form acylceramide which is part of the natural skin barrier.

The first functional studies were conducted with yeast microsomes recombinantly expressing CYP4F22. It was assumed that CYP4F22 catalyses the formation of epoxy alcohols (HEETs) and epoxides (EETs) from arachidonic acid [95]. However, CYP4F22 only poorly metabolized arachidonic acid to 18-HETE which is the 18-hydroxylated arachidonic derivative. Other hypotheses focused on its potential to 20-hydroxylate hepoxilin and trioxilin which were thought to contribute to skin hydration [94]. Then in 2015, a compelling study was published ultimately revealing the function of CYP4F22 [16]. Using recombinant expression systems, CYP4F22 was identified to perform the ω-hydroxylation of ultra-long-chain fatty acids of chain lengths of up to 30 carbons. These ω-hydroxylated fatty acids then condense with sphingolipid long-chain bases to form ultra-long-chain ceramides which in a final step produce acylceramides with linoleic acid (figure 6). Acylceramides are indispensable for a healthy skin barrier formation and to sequester proteins in the skin.

Mutations in the CYP4F22 gene locus have been previously identified in Italian, Spanish, and Chinese patients with congenital ichthyosis. Previously, there were a total of seven mutations known, five of which led to amino acid substitutions as confirmed in Chinese and Spanish cohort studies (F59L, R243H, R372W, H435Y and H436D) [96,97]. In an Italian cohort study, 16.2% of patients with congenital ichthyosis had a mutation in the CYP4F22 gene locus. Three new mutations were identified leading to the amino acid exchanges K128N, R108P, and R515L [98]. Since CYP4F22 has only recently drawn attention for its involvement in congenital ichthyosis, a rise in identified mutations is to be expected which will also lead to more thorough structural and functional studies of this important key player in skin health.

### 6. The future potential of CYP4F enzymes

#### 6.1. In-depth enzymatic studies on CYP4F enzymes are missing

Even though the CYP4F enzyme family has a significant impact on human health and disease, only limited studies are available probing enzyme function and regulation. This is largely due to the fact that, so far, the only protocol available for the generation of recombinant enzyme in *E. coli* was reported for CYP4F11 only and none of the other isoforms [84]. However, highly pure and isolated protein is needed to conduct in-depth structural and functional studies. In terms of substrate scope, we know from the limited studies available that CYP4F isoforms can have a flexible active site, catalyse reactions other than a fatty acid ω-hydroxylation, and, thus, might have a much broader substrate portfolio. Hence, their biological function might be much more diverse with regard to the high CYP4F abundance in the liver and the potential to metabolize xenobiotics.

**Figure 7.**
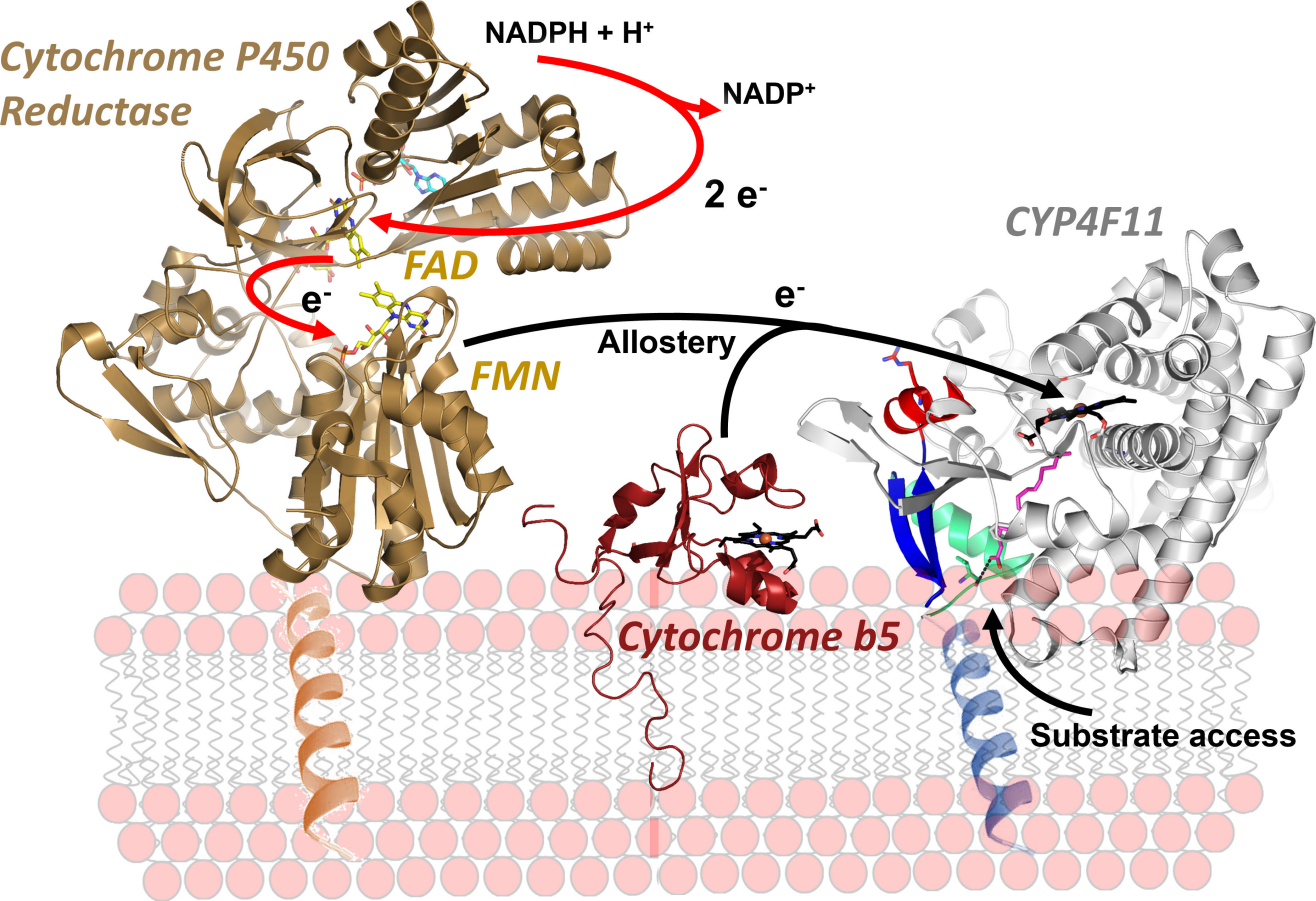
Microsomal P450 redox system with the FAD domain of CPR (brown; PDB: 3QFC) abstracting two electrons from NADPH which are then transferred to the FMN domain. FMN domain then transfers one electron at a time to the P450 4F11 (grey). CYB5 (red; PDB: 2I96) might allosterically regulate substrate binding and turnover. The allosteric influence of these redox partner proteins on CYP4F enzymes has never been systematically studied.

There is no study available that systematically interrogates the interaction with and regulation by redox partner proteins. It is long known that the small heme protein cytochrome b5 (CYB5) can allosterically enhance or inhibit P450 catalysis that is largely isoform- and even substrate-dependent [23]. CYB5 action is even mandatory for the last step of testosterone biosynthesis which is catalysed by the P450 enzymes CYP17A1 [99]. The CPR which delivers electrons to the P450 required for the reaction has long been known as electron transfer protein only (figure 7). However, in recent years, the CPR gained attention as potent allosteric modulator of P450 substrate binding and catalytic activity for various drug metabolizing and steroidogenic P450 enzymes [24]. Nothing is known about the interaction of CYP4F isoforms with these redox proteins. It is particularly striking that an amino acid stretch known to interact with redox partners is highly diverse among CYP4F enzymes which implicates an isoform specific differential interaction with and regulation by redox proteins (figure 1).

#### 6.2. We have little information about CYP4F structure

The lack of protocols for recombinant CYP4F protein also impedes structural studies. Since CYP4F11 exhibits a remarkably different substrate portfolio compared to other ω-hydroxylases, we assume CYP4F11 has a larger substrate entry channel and more flexible active site compared to other CYP4Fs. The Strobel group reported this unique structural feature using a homology model comparing CYP4F11 with CYP4F3A. The broader and more open substrate access channel enables CYP4F11 to accommodate larger molecules, such as erythromycin [10]. Furthermore, the distinct properties of the F/G loop—including variations in residue size, charge, and hydrophobicity—enhance its flexibility in binding diverse substrates. These characteristics set CYP4F11 apart as a unique member of the CYP4F subfamily of P450 ω-hydroxylases. Despite these structural insights, the lack of an experimentally determined crystal structure for CYP4F11—and indeed for any human CYP4 ω-hydroxylases—remains a significant challenge. Due to the inherent difficulties in expressing recombinant membrane proteins, no crystallographic data are currently available. As noted in the introduction, a computed structural model of CYP4F11 was published in 2024, based on the crystal structure of rabbit CYP4B1—the only CYP4 enzyme with an available structure—and the Alphafold model for human CYP4F11 [100]. Determining the crystal structure of any CYP4F enzyme would be highly valuable for researchers in the P450 field, offering crucial insights not only into enzyme architecture but also into structure–activity relationships, ultimately advancing our understanding of CYP4Fs and paving the way for directed drug design.

#### 6.3. CYP4F enzymes in human health and disease

Undoubtedly, the CYP4F enzymes play a major role in the metabolism of fatty acids, vitamins and xenobiotics. They also play a crucial part in the development of human diseases, such as various cancers and hypertension, which is mainly attributed to one of the major CYP4F products, 20-HETE. Usually, 20-HETE controls blood pressure and angiogenesis, but also has detrimental effects when excessively produced. Here, 20-HETE production leads to cancer cell proliferation, tumour angiogenesis, cardiovascular disease, and worsens the outcome of patients with traumatic brain injury [101–103]. The only 20-HETE receptor identified so far is the G-protein coupled receptor (GPCR) GPR75 which has a low nanomolar dissociation constant for 20-HETE [104–106]. The only relevant inhibitor for 20-HETE production so far is the small molecule HET0016 (*N*-(4-butyl-2-methylphenyl)-*N*′-hydroxymethanimidamide). However, HET0016 is an unselective pan-inhibitor of all CYP4 enzymes and also exhibits off-target effects [18]. To promote the use of CYP4F enzymes as drug targets, it is mandatory to identify the involvement of single isoforms for directed targeting and understand the underlying cellular mechanism. Recent research has been focused on deciphering the role of CYP4F enzymes in various disease states. A comprehensive review about the abundance and potential role of individual CYP4F enzymes in various cancers is available [107]. In addition, metabolic dysfunction-associated steatotic liver disease was also associated with CYP4 enzymes, and a comprehensive review can be found in [108] which also provides a comprehensive breakdown of known CYP4F substrates [108].

In summary, although CYP4F enzymes have been of interest for more than two decades, we still have only limited information on their cellular function, enzymatic properties, and structural features. We hope that this review underlines the importance of this enzyme group and that future research will elucidate their role in human health with potential new therapeutic approaches for human disease.

## Data Availability

This article has no additional data.

## References

[1] Guengerich FP. 2019 Cytochrome P450 research and the Journal of Biological Chemistry. J. Biol. Chem. **294**, 1671–1680. (10.1074/jbc.tm118.004144) 29871932 PMC6364786

[2] Edson KZ, Rettie AE. 2013 CYP4 enzymes as potential drug targets: focus on enzyme multiplicity, inducers and inhibitors, and therapeutic modulation of 20-hydroxyeicosatetraenoic acid (20-HETE) synthase and fatty acid ω-hydroxylase activities. Curr. Top. Med. Chem. **13**, 1429–1440. (10.2174/15680266113139990110) 23688133 PMC4245146

[3] Kalsotra A, Strobel HW. 2006 Cytochrome P450 4F subfamily: at the crossroads of eicosanoid and drug metabolism. Pharmacol. Ther. **112**, 589–611. (10.1016/j.pharmthera.2006.03.008) 16926051

[4] Hannemann F, Bichet A, Ewen KM, Bernhardt R. 2007 Cytochrome P450 systems—biological variations of electron transport chains. Biochim. Biophys. Acta **1770**, 330–344. (10.1016/j.bbagen.2006.07.017) 16978787

[5] Zhang C, Catucci G, Di Nardo G, Gilardi G. 2020 Effector role of cytochrome P450 reductase for androstenedione binding to human aromatase. Int. J. Biol. Macromol. **164**, 510–517. (10.1016/j.ijbiomac.2020.07.163) 32698066

[6] Kirischian NL, Wilson JY. 2012 Phylogenetic and functional analyses of the cytochrome P450 family 4. Mol. Phylogenetics Evol. **62**, 458–471. (10.1016/j.ympev.2011.10.016) 22079551

[7] Nelson DR, Goldstone JV, Stegeman JJ. 2013 The cytochrome P450 genesis locus: the origin and evolution of animal cytochrome P450s. Phil. Trans. R. Soc. B **368**, 20120474. (10.1098/rstb.2012.0474) 23297357 PMC3538424

[8] Richard-St-Hilaire A, Gamache I, Pelletier J, Grenier JC, Poujol R, Hussin JG. 2024 Signatures of co-evolution and co-regulation in the CYP3A and CYP4F genes in humans. Genome Biol. Evol. **16**, evad236. (10.1093/gbe/evad236) 38207129 PMC10805436

[9] Chen X *et al*. 2022 CYP4F2-catalyzed metabolism of arachidonic acid promotes stromal cell-mediated immunosuppression in non-small cell lung cancer. Cancer Res. **82**, 4016–4030. (10.1158/0008-5472.can-21-4029) 36006988

[10] Kalsotra A. 2004 Expression and characterization of human cytochrome P450 4F11: putative role in the metabolism of therapeutic drugs and eicosanoids. Toxicol. Appl. Pharmacol. **199**, 295–304. (10.1016/j.taap.2003.12.033) 15364545

[11] Christmas P *et al*. 2001 Alternative splicing determines the function of CYP4F3 by switching substrate specificity. J. Biol. Chem. **276**, 38166–38172. (10.1074/jbc.m104818200) 11461919

[12] Wu CC, Gupta T, Garcia V, Ding Y, Schwartzman ML. 2014 20-HETE and blood pressure regulation: clinical implications. Cardiol. Rev. **22**, 1–12. (10.1097/CRD.0b013e3182961659) 23584425 PMC4292790

[13] Edson KZ, Prasad B, Unadkat JD, Suhara Y, Okano T, Guengerich FP, Rettie AE. 2013 Cytochrome P450-dependent catabolism of vitamin K: ω-hydroxylation catalyzed by human CYP4F2 and CYP4F11. Biochemistry **52**, 8276–8285. (10.1021/bi401208m) 24138531 PMC3900248

[14] Cui X, Nelson DR, Strobel HW. 2000 A novel human cytochrome P450 4F isoform (CYP4F11): cDNA cloning, expression, and genomic structural characterization. Genomics **68**, 161–166. (10.1006/geno.2000.6276) 10964514

[15] Dhar M, Sepkovic DW, Hirani V, Magnusson RP, Lasker JM. 2008 Omega oxidation of 3-hydroxy fatty acids by the human CYP4F gene subfamily enzyme CYP4F11. J. Lipid Res. **49**, 612–624. (10.1194/jlr.m700450-jlr200) 18065749

[16] Ohno Y *et al*. 2015 Essential role of the cytochrome P450 CYP4F22 in the production of acylceramide, the key lipid for skin permeability barrier formation. Proc. Natl Acad. Sci. USA **112**, 7707–7712. (10.1073/pnas.1503491112) 26056268 PMC4485105

[17] Michaels S, Wang MZ. 2014 The revised human liver cytochrome P450 ‘Pie’: absolute protein quantification of CYP4F and CYP3A enzymes using targeted quantitative proteomics. Drug Metab. Dispos. **42**, 1241–1251. (10.1124/dmd.114.058040) 24816681 PMC4109210

[18] Jia H, Brixius B, Bocianoski C, Ray S, Koes DR, Brixius-Anderko S. 2023 Deciphering the role of fatty acid-metabolizing CYP4F11 in lung cancer and its potential as a drug target. Drug Metab. Dispos. **52**, 69–79. (10.1124/dmd.123.001463) 37973374

[19] Hirani V, Yarovoy A, Kozeska A, Magnusson RP, Lasker JM. 2008 Expression of CYP4F2 in human liver and kidney: assessment using targeted peptide antibodies. Arch. Biochem. Biophys. **478**, 59–68. (10.1016/j.abb.2008.06.025) 18662666 PMC2647861

[20] Hsu MH, Baer BR, Rettie AE, Johnson EF. 2017 The crystal structure of cytochrome P450 4B1 (CYP4B1) monooxygenase complexed with octane discloses several structural adaptations for ω-hydroxylation. J. Biol. Chem. **292**, 5610–5621. (10.1074/jbc.m117.775494) 28167536 PMC5392703

[21] Jennings GK, Hsu MH, Shock LS, Johnson EF, Hackett JC. 2018 Noncovalent interactions dominate dynamic heme distortion in cytochrome P450 4B1. J. Biol. Chem. **293**, 11433–11446. (10.1074/jbc.ra118.004044) 29858244 PMC6065186

[22] Kandel SE, Lampe JN. 2014 Role of protein–protein interactions in cytochrome P450-mediated drug metabolism and toxicity. Chem. Res. Toxicol. **27**, 1474–1486. (10.1021/tx500203s) 25133307 PMC4164225

[23] Bart AG, Scott EE. 2017 Structural and functional effects of cytochrome b5 interactions with human cytochrome P450 enzymes. J. Biol. Chem. **292**, 20818–20833. (10.1074/jbc.RA117.000220) 29079577 PMC5743060

[24] Burris-Hiday SD, Scott EE. 2023 Allosteric modulation of cytochrome P450 enzymes by the NADPH cytochrome P450 reductase FMN-containing domain. J. Biol. Chem. **299**, 105112. (10.1016/j.jbc.2023.105112) 37517692 PMC10481364

[25] Brixius-Anderko S, Scott EE. 2021 Structural and functional insights into aldosterone synthase interaction with its redox partner protein adrenodoxin. J. Biol. Chem. **296**, 100794. (10.1016/j.jbc.2021.100794) 34015331 PMC8215293

[26] Lasker JM, Chen WB, Wolf I, Bloswick BP, Wilson PD, Powell PK. 2000 Formation of 20-hydroxyeicosatetraenoic acid, a vasoactive and natriuretic eicosanoid, in human kidney. J. Biol. Chem. **275**, 4118–4126. (10.1074/jbc.275.6.4118) 10660572

[27] Fer M, Corcos L, Dréano Y, Plée-Gautier E, Salaün JP, Berthou F, Amet Y. 2008 Cytochromes P450 from family 4 are the main omega hydroxylating enzymes in humans: CYP4F3B is the prominent player in PUFA metabolism. J. Lipid Res. **49**, 2379–2389. (10.1194/jlr.m800199-jlr200) 18577768

[28] Kikuta Y, Kusunose E, Kondo T, Yamamoto S, Kinoshita H, Kusunose M. 1994 Cloning and expression of a novel form of leukotriene B4 omega-hydroxylase from human liver. FEBS Lett. **348**, 70–74. (10.1016/0014-5793(94)00587-7) 8026587

[29] Powell PK, Wolf I, Jin R, Lasker JM. 1998 Metabolism of arachidonic acid to 20-hydroxy-5,8,11,14-eicosatetraenoic acid by P450 enzymes in human liver: involvement of CYP4F2 and CYP4A11. J. Pharmacol. Exp. Ther. **285**, 1327–1336. (10.1016/S0022-3565(24)37530-5) 9618440

[30] Kikuta Y, Kusunose E, Kusunose M. 2000 Characterization of human liver leukotriene B4-hydroxylase P450 (CYP4F2). J. Biochem. **127**, 1047–1052. (10.1093/oxfordjournals.jbchem.a022696) 10833273

[31] Wang MZ, Wu JQ, Bridges AS, Zeldin DC, Kornbluth S, Tidwell RR, Hall JE, Paine MF. 2007 Human enteric microsomal CYP4F enzymes O-demethylate the antiparasitic prodrug pafuramidine. Drug Metab. Dispos. **35**, 2067–2075. (10.1124/dmd.107.016428) 17709372 PMC2364724

[32] Rochat B, Zoete V, Grosdidier A, von Grünigen S, Marull M, Michielin O. 2008 In vitro biotransformation of imatinib by the tumor expressed CYP1A1 and CYP1B1. Biopharm. Drug Dispos. **29**, 103–118. (10.1002/bdd.598) 18188833

[33] Jin Y, Zollinger M, Borell H, Zimmerlin A, Patten CJ. 2011 CYP4F enzymes are responsible for the elimination of fingolimod (FTY720), a novel treatment of relapsing multiple sclerosis. Drug Metab. Dispos. **39**, 191–198. (10.1124/dmd.110.035378) 21045201

[34] Seguin RP, Herron JM, Lopez VA, Dempsey JL, Xu L. 2019 Metabolism of benzalkonium chlorides by human hepatic cytochromes P450. Chem. Res. Toxicol. **32**, 2466–2478. (10.1021/acs.chemrestox.9b00293) 31730751 PMC7269367

[35] Xue Y, Hieda Y, Saito Y, Nomura T, Fujihara J, Takayama K, Kimura K, Takeshita H. 2004 Distribution and disposition of benzalkonium chloride following various routes of administration in rats. Toxicol. Lett. **148**, 113–123. (10.1016/j.toxlet.2003.12.068) 15019095

[36] Zhang X, Hardwick JP. 2000 Regulation of CYP4F2 leukotriene B4 ω-hydroxylase by retinoic acids in HepG2 cells. Biochem. Biophys. Res. Commun. **279**, 864–871. (10.1006/bbrc.2000.4020) 11162441

[37] Hsu MH, Savas U, Griffin KJ, Johnson EF. 2007 Regulation of human cytochrome P450 4F2 expression by sterol regulatory element-binding protein and lovastatin. J. Biol. Chem. **282**, 5225–5236. (10.1074/jbc.M608176200) 17142457

[38] Hsu MH, Savas Ü, Lasker JM, Johnson EF. 2011 Genistein, resveratrol, and 5-aminoimidazole-4-carboxamide-1-β-d-ribofuranoside induce cytochrome P450 4F2 expression through an AMP-activated protein kinase-dependent pathway. J. Pharmacol. Exp. Ther. **337**, 125–136. (10.1124/jpet.110.175851) 21205922 PMC3063743

[39] Cooper GM *et al*. 2008 A genome-wide scan for common genetic variants with a large influence on warfarin maintenance dose. Blood **112**, 1022–1027. (10.1182/blood-2008-01-134247) 18535201 PMC2515139

[40] Caldwell MD *et al*. 2008 CYP4F2 genetic variant alters required warfarin dose. Blood **111**, 4106–4112. (10.1182/blood-2007-11-122010) 18250228 PMC2288721

[41] McDonald MG, Rieder MJ, Nakano M, Hsia CK, Rettie AE. 2009 CYP4F2 is a vitamin K1 oxidase: an explanation for altered warfarin dose in carriers of the V433M variant. Mol. Pharmacol. **75**, 1337–1346. (10.1124/mol.109.054833) 19297519 PMC2684883

[42] Danese E *et al*. 2012 Impact of the CYP4F2 p.V433M polymorphism on coumarin dose requirement: systematic review and meta-analysis. Clin. Pharmacol. Ther. **92**, 746–756. (10.1038/clpt.2012.184) 23132553 PMC3731755

[43] Zubiaur P *et al*. 2024 PharmVar GeneFocus: CYP4F2. Clin. Pharmacol. Ther. **116**, 963–975. (10.1002/cpt.3405) 39135485 PMC11452279

[44] Takeuchi F *et al*. 2009 A genome-wide association study confirms VKORC1, CYP2C9, and CYP4F2 as principal genetic determinants of warfarin dose. PLoS Genet. **5**, e1000433. (10.1371/journal.pgen.1000433) 19300499 PMC2652833

[45] Liang R, Wang C, Zhao H, Huang J, Hu D, Sun Y. 2012 Influence of CYP4F2 genotype on warfarin dose requirement: a systematic review and meta-analysis. Thromb. Res. **130**, 38–44. (10.1016/j.thromres.2011.11.043) 22192158

[46] Scott SA, Khasawneh R, Peter I, Kornreich R, Desnick RJ. 2010 Combined CYP2C9, VKORC1 and CYP4F2 frequencies among racial and ethnic groups. Pharmacogenomics **11**, 781–791. (10.2217/pgs.10.49) 20504253 PMC2904527

[47] Farajzadeh-Dehkordi M, Mafakher L, Samiee-Rad F, Rahmani B. 2023 Computational analysis of missense variant CYP4F2*3 (V433M) in association with human CYP4F2 dysfunction: a functional and structural impact. BMC Mol. Cell Biol. **24**, 17. (10.1186/s12860-023-00479-0) 37161313 PMC10170697

[48] van Engen CE *et al*. 2016 CYP4F2 affects phenotypic outcome in adrenoleukodystrophy by modulating the clearance of very long-chain fatty acids. Biochim. Biophys. Acta **1862**, 1861–1870. (10.1016/j.bbadis.2016.07.006) 27425035

[49] Sontag TJ, Parker RS. 2007 Influence of major structural features of tocopherols and tocotrienols on their omega-oxidation by tocopherol-omega-hydroxylase. J. Lipid Res. **48**, 1090–1098. (10.1194/jlr.M600514-JLR200) 17284776

[50] Bardowell SA, Duan F, Manor D, Swanson JE, Parker RS. 2012 Disruption of mouse cytochrome P450 4f14 (Cyp4f14 gene) causes severe perturbations in vitamin E metabolism. J. Biol. Chem. **287**, 26077–26086. (10.1074/jbc.m112.373597) 22665481 PMC3406691

[51] Jiang Q. 2022 Metabolism of natural forms of vitamin E and biological actions of vitamin E metabolites. Free Radic. Biol. Med. **179**, 375–387. (10.1016/j.freeradbiomed.2021.11.012) 34785321 PMC9018116

[52] Shekhar S, Varghese K, Li M, Fan L, Booz G, Roman R, Fan F. 2019 Conflicting roles of 20-HETE in hypertension and stroke. Int. J. Mol. Sci. **20**, 4500. (10.3390/ijms20184500) 31514409 PMC6770042

[53] Zhang C, Booz GW, Yu Q, He X, Wang S, Fan F. 2018 Conflicting roles of 20-HETE in hypertension and renal end organ damage. Eur. J. Pharmacol. **833**, 190–200. (10.1016/j.ejphar.2018.06.010) 29886242 PMC6057804

[54] Stec DE, Roman RJ, Flasch A, Rieder MJ. 2007 Functional polymorphism in human CYP4F2 decreases 20-HETE production. Physiol. Genom. **30**, 74–81. (10.1152/physiolgenomics.00003.2007) 17341693

[55] Ward NC, Tsai IJ, Barden A, van Bockxmeer FM, Puddey IB, Hodgson JM, Croft KD. 2008 A single nucleotide polymorphism in the CYP4F2 but not CYP4A11 gene Is associated with increased 20-HETE excretion and blood pressure. Hypertension **51**, 1393–1398. (10.1161/HYPERTENSIONAHA.107.104463) 18391101

[56] Munshi A, Sharma V, Kaul S, Al-Hazzani A, Alshatwi AA, Shafi G, Koppula R, Mallemoggala SB, Jyothy A. 2012 Association of 1347 G/A cytochrome P450 4F2 (CYP4F2) gene variant with hypertension and stroke. Mol. Biol. Rep. **39**, 1677–1682. (10.1007/s11033-011-0907-y) 21625857

[57] Fava C, Rosberg L, Lippi G, Hedblad B, Engström G, Berglund G, Minuz P, Melander O *et al*. 2008 The V433M variant of the CYP4F2 is associated with ischemic stroke in male Swedes beyond its effect on blood pressure. Hypertension **52**, 373–380. (10.1161/hypertensionaha.108.114199) 18574070

[58] Ding H *et al*. 2010 Association of common variants of CYP4A11 and CYP4F2 with stroke in the Han Chinese population. Pharmacogenet. Genomics **20**, 187–194. (10.1097/FPC.0b013e328336eefe) 20130494 PMC3932492

[59] Fu Z *et al*. 2008 Haplotype-based case-control study of the human CYP4F2 gene and essential hypertension in Japanese subjects. Hypertens. Res. **31**, 1719–1726. (10.1291/hypres.31.1719) 18971550

[60] Wu Y, Zhao J, Zhao Y, Huang T, Ma X, Pang H, Zhang M. 2019 Genetic variants in CYP4F2 were significantly correlated with susceptibility to ischemic stroke. BMC Med. Genet. **20**, 155. (10.1186/s12881-019-0888-6) 31510945 PMC6737589

[61] Tanzo JT *et al*. 2023 CYP4F2 is a human-specific determinant of circulating N-acyl amino acid levels. J. Biol. Chem. **299**, 104764. (10.1016/j.jbc.2023.104764) 37121548 PMC10318452

[62] Eun HS, Cho SY, Lee BS, Seong IO, Kim KH. 2018 Profiling cytochrome P450 family 4 gene expression in human hepatocellular carcinoma. Mol. Med. Rep. **18**, 4865–4876. (10.3892/mmr.2018.9526) 30280198 PMC6236316

[63] Wan S, Pan Q, Yang G, Kuang J, Luo S. 2020 Role of CYP4F2 as a novel biomarker regulating malignant phenotypes of liver cancer cells via the Nrf2 signaling axis. Oncol. Lett. **20**, 13. (10.3892/ol.2020.11874) 32774486 PMC7405372

[64] Yang J *et al*. 2024 Targeting estrogen mediated CYP4F2/CYP4F11-20-HETE metabolic disorder decelerates tumorigenesis in ER+ breast cancer. Biochem. Biophys. Rep. **38**, 101706. (10.1016/j.bbrep.2024.101706) 38646426 PMC11033080

[65] Shak S, Goldstein IM. 1984 Omega-oxidation is the major pathway for the catabolism of leukotriene B4 in human polymorphonuclear leukocytes. J. Biol. Chem. **259**, 10181–10187. (10.1016/s0021-9258(18)90946-4) 6088485

[66] Powell WS. 1984 Properties of leukotriene B4 20-hydroxylase from polymorphonuclear leukocytes. J. Biol. Chem. **259**, 3082–3089. (10.1016/s0021-9258(17)43263-7) 6321494

[67] Sumimoto J, Takeshige K, Minakami S. 1988 Characterization of human neutrophil leukotriene B4 omega-hydroxylase as a system involving a unique cytochrome P-450 and NADPH-cytochrome P-450 reductase. Eur. J. Biochem. **172**, 315–324. (10.1111/j.1432-1033.1988.tb13889.x) 3127205

[68] Le Quéré V, Plée-Gautier E, Potin P, Madec S, Salaün JP. 2004 Human CYP4F3s are the main catalysts in the oxidation of fatty acid epoxides. J. Lipid Res. **45**, 1446–1458. (10.1194/jlr.M300463-JLR200) 15145985

[69] Christmas P, Ursino SR, Fox JW, Soberman RJ. 1999 Expression of the CYP4F3 gene. Tissue-specific splicing and alternative promoters generate high and low K(m) forms of leukotriene B₄ ω-hydroxylase. J. Biol. Chem. **274**, 21191–21199. (10.1074/jbc.274.30.21191) 10409674

[70] Christmas P, Carlesso N, Shang H, Cheng SM, Weber BM, Preffer FI, Scadden DT, Soberman RJ. 2003 Myeloid expression of cytochrome P450 4F3 is determined by a lineage-specific alternative promoter. J. Biol. Chem. **278**, 25133–25142. (10.1074/jbc.m302106200) 12709424

[71] Mizukami Y, Sumimoto H, Takeshige K. 2004 Induction of cytochrome CYP4F3A in all-trans-retinoic acid-treated HL60 cells. Biochem. Biophys. Res. Commun. **315**, 104–109. (10.1016/j.bbrc.2004.01.132) 14715252

[72] Xu Z, Xu C, Lu J, He C, Wang X, Zhu D, Wang A, Zhang Z, Jiang C. 2024 Cytochrome P450 F3 promotes colorectal cancer via inhibiting NRF2-mediated ferroptosis. Transl. Oncol. **48**, 102077. (10.1016/j.tranon.2024.102077) 39106550 PMC11357859

[73] Antoun J, Goulitquer S, Amet Y, Dreano Y, Salaun JP, Corcos L, Plée-Gautier E. 2008 CYP4F3B is induced by PGA1 in human liver cells: a regulation of the 20-HETE synthesis. J. Lipid Res. **49**, 2135–2141. (10.1194/jlr.m800043-jlr200) 18566475

[74] Plée-Gautier E, Antoun J, Goulitquer S, Le Jossic-Corcos C, Simon B, Amet Y, Salaün JP, Corcos L. 2012 Statins increase cytochrome P450 4F3-mediated eicosanoids production in human liver cells: a PXR dependent mechanism. Biochem. Pharmacol. **84**, 571–579. (10.1016/j.bcp.2012.05.012) 22634049

[75] Unoki M *et al*. 2000 Association studies of 33 single nucleotide polymorphisms (SNPs) in 29 candidate genes for bronchial asthma: positive association of a T924C polymorphism in the thromboxane A2 receptor gene. Hum. Genet. **106**, 440–446. (10.1007/s004390000267) 10830912

[76] Curley CR, Monsuur AJ, Wapenaar MC, Rioux JD, Wijmenga C. 2006 A functional candidate screen for coeliac disease genes. Eur. J. Hum. Genet. **14**, 1215–1222. (10.1038/sj.ejhg.5201687) 16835590

[77] Costea I *et al*. 2010 Genes involved in the metabolism of poly-unsaturated fatty-acids (PUFA) and risk for Crohn’s disease in children & young adults. PLoS One **5**, e15672. (10.1371/journal.pone.0015672) 21187935 PMC3004960

[78] Costea I, Mack DR, Lemaitre RN, Israel D, Marcil V, Ahmad A, Amre DK. 2014 Interactions between the dietary polyunsaturated fatty acid ratio and genetic factors determine susceptibility to pediatric Crohn’s disease. Gastroenterology **146**, 929–931.(10.1053/j.gastro.2013.12.034) 24406470

[79] Ongun MC, Tonyali NV, Kaplan O, Deger I, Celebier M, Basci Akduman NE, Sahin D, Yucel A, Babaoglu MO. 2023 Effects of genetic polymorphisms of CYP2J2, CYP2C9, CYP2C19, CYP4F2, CYP4F3 and CYP4A11 enzymes in preeclampsia and gestational hypertension. Placenta **137**, 88–95. (10.1016/j.placenta.2023.04.019) 37141740

[80] Yin J *et al*. 2017 Pathway‐analysis of published genome‐wide association studies of lung cancer: a potential role for the CYP4F3 locus. Mol. Carcinog. **56**, 1663–1672. (10.1002/mc.22622) 28150878 PMC5423820

[81] Ananthakrishnan AN, Khalili H, Song M, Higuchi LM, Lochhead P, Richter JM, Chan AT. 2017 Genetic polymorphisms in fatty acid metabolism modify the association between dietary n3:n6 intake and risk of ulcerative colitis: a prospective cohort study. Inflamm. Bowel Dis. **23**, 1898–1904. (10.1097/MIB.0000000000001236) 28991856 PMC5675119

[82] Smeets E *et al*. 2022 A disease‐associated missense mutation in CYP4F3 affects the metabolism of leukotriene B4 via disruption of electron transfer. J. Cachexia Sarcopenia Muscle **13**, 2242–2253. (10.1002/jcsm.13022) 35686338 PMC9397552

[83] Johnson AL, Edson KZ, Totah RA, Rettie AE. 2015 Cytochrome P450 ω-hydroxylases in inflammation and cancer. Adv. Pharmacol. **74**, 223–262. (10.1016/bs.apha.2015.05.002) 26233909 PMC4667791

[84] Tang Z, Salamanca-Pinzón SG, Wu ZL, Xiao Y, Guengerich FP. 2010 Human cytochrome P450 4F11: heterologous expression in bacteria, purification, and characterization of catalytic function. Arch. Biochem. Biophys. **494**, 86–93. (10.1016/j.abb.2009.11.017) 19932081 PMC2812615

[85] Skorokhod O, Triglione V, Barrera V, Di Nardo G, Valente E, Ulliers D, Schwarzer E, Gilardi G. 2023 Posttranslational modification of human cytochrome CYP4F11 by 4-hydroxynonenal impairs ω-hydroxylation in malaria pigment hemozoin-fed monocytes: the role in malaria immunosuppression. Int. J. Mol. Sci. **24**, 10232. (10.3390/ijms241210232) 37373382 PMC10299294

[86] Robb CS, Reisky L, Bornscheuer UT, Hehemann JH. 2018 Specificity and mechanism of carbohydrate demethylation by cytochrome P450 monooxygenases. Biochem. J. **475**, 3875–3886. (10.1042/bcj20180762) 30404923 PMC6292453

[87] Theodoropoulos PC *et al*. 2016 Discovery of tumor-specific irreversible inhibitors of stearoyl CoA desaturase. Nat. Chem. Biol. **12**, 218–225. (10.1038/nchembio.2016) 26829472 PMC4798879

[88] Winterton SE, Capota E, Wang X, Chen H, Mallipeddi PL, Williams NS, Posner BA, Nijhawan D, Ready JM. 2018 Discovery of cytochrome P450 4F11 activated inhibitors of stearoyl coenzyme A desaturase. J. Med. Chem. **61**, 5199–5221. (10.1021/acs.jmedchem.8b00052) 29869888 PMC6350083

[89] Wang Y, Bell JC, Keeney DS, Strobel HW. 2010 Gene regulation of CYP4F11 in human keratinocyte HaCaT cells. Drug Metab. Dispos. **38**, 100–107. (10.1124/dmd.109.029025) 19812349 PMC2802424

[90] Mangelsdorf DJ, Evans RM. 1995 The RXR heterodimers and orphan receptors. Cell **83**, 841–850. (10.1016/0092-8674(95)90200-7) 8521508

[91] Bell JC, Strobel HW. 2012 Regulation of cytochrome P450 4F11 by nuclear transcription factor-κB. Drug Metab. Dispos. **40**, 205–211. (10.1124/dmd.111.041178) 22011441 PMC3250053

[92] Zhang T, Zhan Z, Chen Y, Chen J, Han W, Liang Z, Liu Q, Liu S, Tang L. 2021 Regulation of cytochrome P450 4F11 expression by liver X receptor alpha. Int. Immunopharmacol. **90**, 107240. (10.1016/j.intimp.2020.107240) 33310663

[93] Bar-Peled L *et al*. 2017 Chemical proteomics identifies druggable vulnerabilities in a genetically defined cancer. Cell **171**, 696–709.(10.1016/j.cell.2017.08.051) 28965760 PMC5728659

[94] Kelly EJ, Nakano M, Rohatgi P, Yarov-Yarovoy V, Rettie AE. 2011 Finding homes for orphan cytochrome P450s: CYP4V2 and CYP4F22 in disease states. Mol. Interv. **11**, 124–132. (10.1124/mi.11.2.10) 21540472 PMC3109859

[95] Nilsson T, Ivanov IV, Oliw EH. 2010 LC–MS/MS analysis of epoxyalcohols and epoxides of arachidonic acid and their oxygenation by recombinant CYP4F8 and CYP4F22. Arch. Biochem. Biophys. **494**, 64–71. (10.1016/j.abb.2009.11.013) 19919823

[96] Tang H, Shi X, Zhang G. 2021 Novel compound heterozygous mutations in the CYP4F22 gene in a patient with autosomal recessive congenital ichthyosis. Clin. Case Rep. **9**, e05082. (10.1002/ccr3.5082) 34917360 PMC8645175

[97] Esperón-Moldes U *et al*. 2020 Novel CYP4F22 mutations associated with autosomal recessive congenital ichthyosis (ARCI). Study of the CYP4F22 c.1303C>T founder mutation. PLoS One **15**, e0229025. (10.1371/journal.pone.0229025) 32069299 PMC7028276

[98] Diociaiuti A *et al*. 2024 Cross-sectional study on autosomal recessive congenital ichthyoses: association of genotype with disease severity, phenotypic, and ultrastructural features in 74 Italian patients. Dermatology **240**, 1–17. (10.1159/000536366) 38588653 PMC11168449

[99] Peng HM, Im SC, Pearl NM, Turcu AF, Rege J, Waskell L, Auchus RJ. 2016 Cytochrome b₅ activates the 17,20-lyase activity of human cytochrome P450 17A1 by increasing the coupling of NADPH consumption to androgen production. Biochemistry **55**, 4356–4365. (10.1021/acs.biochem.6b00532) 27426448 PMC5287367

[100] Jia H, Brixius B, Bocianoski C, Ray S, Koes DR, Brixius-Anderko S. 2024 Deciphering the role of fatty acid-metabolizing CYP4F11 in lung cancer and its potential as a drug target. Drug Metab. Dispos. **52**, 69–79. (10.1124/dmd.123.001463) 37973374

[101] Cui W *et al*. 2021 20‐HETE synthesis inhibition attenuates traumatic brain injury-induced mitochondrial dysfunction and neuronal apoptosis via the SIRT1/PGC‐1α pathway: a translational study. Cell Prolif. **54**, e12964. (10.1111/cpr.12964) 33314534 PMC7848954

[102] Alexanian A, Miller B, Roman RJ, Sorokin A. 2012 20-HETE-producing enzymes are up-regulated in human cancers. Cancer Genom. Proteom. **9**, 163–169. PMC3601443 22798501

[103] Garcia V *et al*. 2017 20-HETE signals through G-Protein-Coupled Receptor GPR75 (G_q_) to affect vascular function and trigger hypertension. Circ. Res. **120**, 1776–1788. (10.1161/CIRCRESAHA.116.310525) 28325781 PMC5446268

[104] Cárdenas S, Colombero C, Panelo L, Dakarapu R, Falck JR, Costas MA, Nowicki S. 2020 GPR75 receptor mediates 20-HETE-signaling and metastatic features of androgen-insensitive prostate cancer cells. Biochim. Biophys. Acta **1865**, 158573. (10.1016/j.bbalip.2019.158573) PMC6957769 31760076

[105] Fan F, Roman RJ. 2017 GPR75 identified as the first 20-HETE receptor: a chemokine receptor adopted by a new family. Circ. Res. **120**, 1696–1698. (10.1161/CIRCRESAHA.117.311022) 28546348 PMC5766006

[106] Froogh G, Garcia V, Laniado Schwartzman M. 2022 The CYP/20-HETE/GPR75 axis in hypertension. Adv. Pharmacol. **94**, 1–25. (10.1016/bs.apha.2022.02.003) 35659370 PMC10123763

[107] Evangelista EA, Cho CW, Aliwarga T, Totah RA. 2020 Expression and function of eicosanoid-producing cytochrome P450 enzymes in solid tumors. Front. Pharmacol. **11**, 828. (10.3389/fphar.2020.00828) 32581794 PMC7295938

[108] Leahy C *et al*. 2024 The fatty acid omega hydroxylase genes (CYP4 family) in the progression of metabolic dysfunction-associated steatotic liver disease (MASLD): an RNA sequence database analysis and review. Biochem. Pharmacol. **228**, 116241. (10.1016/j.bcp.2024.116241) 38697309 PMC11774579

